# WESSBAS: extraction of probabilistic workload specifications for load testing and performance prediction—a model-driven approach for session-based application systems

**DOI:** 10.1007/s10270-016-0566-5

**Published:** 2016-10-20

**Authors:** Christian Vögele, André van Hoorn, Eike Schulz, Wilhelm Hasselbring, Helmut Krcmar

**Affiliations:** 1 fortiss GmbH, 80805 Munich, Germany; 2 Institute of Software Technology, University of Stuttgart, 70569 Stuttgart, Germany; 3 ARIVA.DE AG, 24118 Kiel, Germany; 4 Department of Computer Science, Kiel University, 24118 Kiel, Germany; 5 Chair for Information Systems, Technical University of Munich (TUM), 85748 Garching, Germany

**Keywords:** Workload specifications, Load testing, Performance prediction, Performance models

## Abstract

The specification of workloads is required in order to evaluate performance characteristics of application systems using load testing and model-based performance prediction. Defining workload specifications that represent the real workload as accurately as possible is one of the biggest challenges in both areas. To overcome this challenge, this paper presents an approach that aims to automate the extraction and transformation of workload specifications for load testing and model-based performance prediction of session-based application systems. The approach (WESSBAS) comprises three main components. First, a system- and tool-agnostic domain-specific language (DSL) allows the layered modeling of workload specifications of session-based systems. Second, instances of this DSL are automatically extracted from recorded session logs of production systems. Third, these instances are transformed into executable workload specifications of load generation tools and model-based performance evaluation tools. We present transformations to the common load testing tool Apache JMeter and to the Palladio Component Model. Our approach is evaluated using the industry-standard benchmark SPECjEnterprise2010 and the World Cup 1998 access logs. Workload-specific characteristics (e.g., session lengths and arrival rates) and performance characteristics (e.g., response times and CPU utilizations) show that the extracted workloads match the measured workloads with high accuracy.

## Introduction

The specification and execution of workloads is essential for evaluating performance properties of application systems. In order to assess whether non-functional performance requirements of these systems can be met, load testing and model-based performance evaluation approaches are applied [14, 58]. Workload specifications serve as input for load testing to generate synthetic workload to the (SUT), i.e., executing a set of customer requests [23, 32, 38]. Additionally, several specifications are taken into account in formalisms for model-based performance evaluation approaches, to predict performance properties early in the software development cycle [8, 31, 58].

In session-based application systems, especially Web-based systems, different types of users interact with the system in a sequence of interdependent requests. The complexity of these interactions makes the workload specification a difficult task [37]. Thus, the manual creation of these workload specifications is time consuming [3] and error prone [47]. One of the main challenges is to ensure that these specifications are representative compared to the real workload [21]. This is a key requirement for both load testing and model-based performance prediction approaches. To ensure that the measured and the predicted performance characteristics of the SUT are comparable, similar workload specifications must be used. However, there is a lack of approaches enabling the common automatic extraction and specification of workloads for both approaches. The extraction and specification of workloads is done separately for each approach and each tool which results in additional specification and maintenance effort. The reasons for this development are that these approaches are not integrated and that workload specifications are defined on different levels of detail. Measurement-based approaches need detailed system-specific information like protocol data, whereas model-based approaches are often specified on a more abstract level.

In response to these challenges, this paper presents our WESSBAS^1^ approach for specifying and extracting representative workloads for session-based application systems. We introduce a (DSL), called WESSBAS-DSL, which enables the system- and tool-agnostic modeling of these workload specifications. Recorded session logs are used as a basis for the automatic extraction of WESSBAS-DSL instances. Different groups of customers showing similar navigational patterns are identified during the creation of these instances. Additionally, inter-request dependencies (Guards and Actions (GaAs)) among the execution of requests are automatically learned. These dependencies come from the fact that the execution of requests often depends on the result of previous requests. The combination of probabilities and GaAs requires the calculation of conditional probabilities, which are also determined in an automatic way. Finally, protocol information required to generate executable load tests are integrated.

**Fig. 1:**
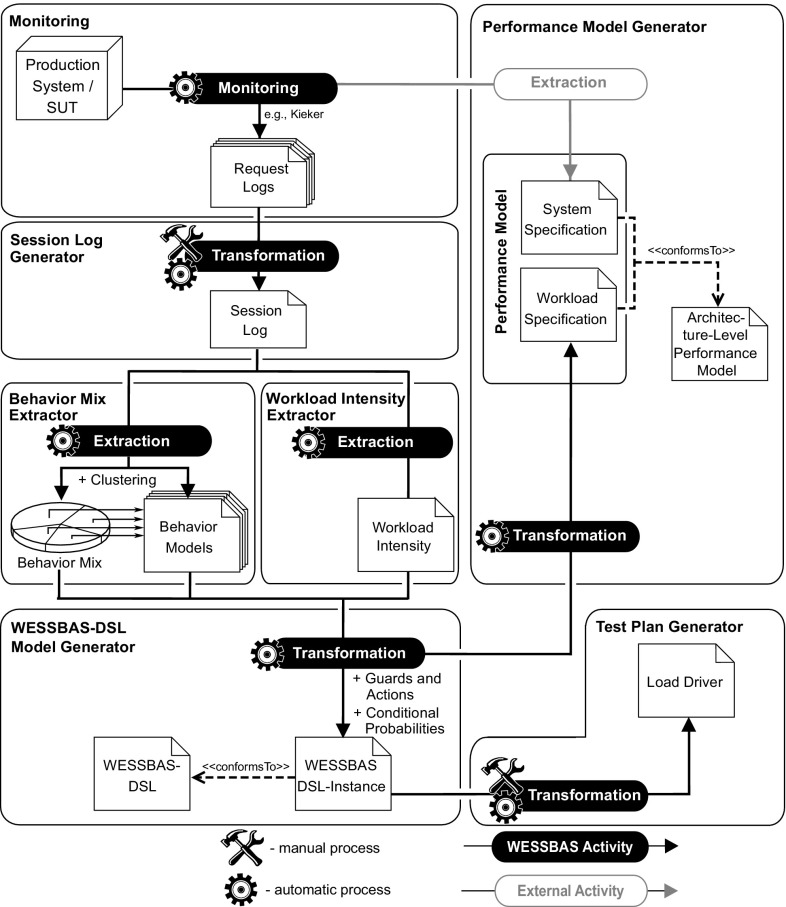
Overview of the WESSBAS approach

As an example, the resulting WESSBAS-DSL instances are then transformed to executable workload specifications for the common load testing tool Apache JMeter, including the Markov4JMeter extension developed in our previous work [50]. Furthermore, the DSL instances are transformed into workload specifications of the Palladio Component Model [8] representing an architecture-level performance modeling language. We focus on architecture-level performance models as they permit to model system architecture, execution environment, and workload specification separately from each other [11]. Figure 1 provides an overview of the WESSBAS approach.

The sharing of a common workload model has several key benefits for both approaches during the system life cycle. During development time for example, system specifications (e.g., Unified Modeling Language (UML) diagrams) or information from expert interviews are available in order to derive estimates about the workload profile. This knowledge can be encoded in the WESSBAS-DSL and then transformed to a performance model to conduct early performance predictions. Once the application system is running and load tests should be executed, parts of the load test (e.g., user scenarios and workload intensity) can be generated based on the WESSBAS-DSL. Thus, the workload only needs to be created once for both approaches.

In order to evaluate the performance characteristics of new releases, load tests are often conducted on test systems. The workload used during these tests should be comparable to the workload of the production systems. This has the advantage that bottlenecks which also occur in production systems can be found with a higher probability. Thus, extracting the workload from the production system and transforming it to load tests and workload specifications of performance models comes with several benefits. The first benefit is that the effort to create and specify load tests and performance models is reduced. Because production workloads can change over time, the latest workload can be extracted again in an easy way to reduce maintenance effort. The second benefit is that the integration of software development and operations (DevOps) is supported [13]. The extracted workload during operations can be used for measurement-based [16] and model-based [12] continuous integration approaches to detect performance regressions during development.

The WESSBAS approach is also useful to validate performance models. To validate the correctness of a performance model, the simulation results are compared with measurement results derived from the system. For the comparability of these results, it must be ensured that the simulation results and the measurements are derived by applying the same workload specification.

To summarize, the contribution of this paper is our WESSBAS approach for automatic extraction of probabilistic workload specifications of session-based application systems comprising the following elements:

1. A DSL for modeling session-based probabilistic workload specifications.
2. An automatic extraction of DSL instances from recorded system logs including the clustering of navigational patterns.
3. Transformations from DSL instances to executable JMeter Test Plans and to workload specifications of the Palladio Component Model (PCM).
4. Tool support for this approach.

To the best of our knowledge, WESSBAS is the first approach to enable the process from runtime data to executable load tests and performance prediction approaches. The tool support serves as an extensible implementation of the approach, including the DSL, the extraction, as well as a proof-of-concept transformation from the DSL to JMeter Test Plans and workload specifications of PCM. The developed tools^2^ and the models and results of this paper [55] are publicly available online.

This paper builds on our previous work [50, 51, 54] on the extraction and specification of workload specifications and contains the following major improvements and extensions:

1. Tool support for the transformation of arbitrary system logs to the required session log format.
2. Automatic learning of GaAs and the calculation of conditional probabilities.
3. Generation of executable load tests (this includes the extraction and integration of protocol information).
4. Comprehensive evaluation of workload characteristics (e.g., session lengths and action counts) and performance characteristics (e.g., response times and CPU utilizations) against the industry-standard benchmark SPECjEnterprise2010 and the World Cup 1998 access logs.

The remainder of this paper is structured as follows: Sect. 2 provides an overview of related work. In Sect. 3, the used workload formalism required to understand the paper is introduced. The extraction of WESSBAS-DSL instances is presented in Sect. 4. The description of the JMeter Test Plan generation process in Sect. 5 is followed by the illustration of the transformation of workload specifications to performance models in Sect. 6. The evaluation of the proposed approach can be found in Sect. 7. Finally, Sect. 8 details conclusions of our work and presents suggestions for future work.

## Related work

Workload specification (also referred to as workload characterization) is defined by the process of first analyzing key characteristics of user interactions (this includes other systems as well) with an application system and modeling these characteristics into a workload model [17, 26]. The key workload characteristics of session-based systems can be divided into intra-session and inter-session metrics [23]. Intra-session metrics characterize single sessions and include the session length, number of requests per session, and think times between the executions of the requests. They also describe the behavior of the user as a sequence of executed requests. In contrast, the inter-session metrics characterize the number of sessions per user and the number of active sessions over time (also referred to as workload intensity).

We group the related work on workload characterization into user behavior modeling and workload intensity. Related work on the extraction of workloads and workload modeling for performance models are also introduced.

### User behavior modeling

User behavior is either specified script-based, or it is specified using analytical models. Scripts representing single-user scenarios with a fixed sequence of user requests are executed by a number of concurrent load generator threads. These scripts are quite easy to record and execute. However, they provide little opportunity to vary workload characteristics, such as different navigational patterns, and therefore are often not as representative as the real workload [21, 35]. Furthermore, as examined by Rodrigues et al. [43], the effort to generate capture and replay scripts is higher with these scripts than the effort using analytical models with increasing complexity of the software system.

In order to model the user behavior in a more representative way, analytical models were introduced. A popular way to model user behavior, especially user behavior related to Web sites, are Markov Chains [60]. An approach similar to our approach was proposed by Menascé et al. [36, 38]. These authors extract so-called Customer Behavior Model Graphs (CBMGs) from HTTP server logs, which are based on Markov Chains. They apply K-means clustering to identify CBMGs for similar types of users. In contrast, in our work, an advancement of the K-means algorithm, called X-means is applied. Another approach using the CBMGs to generate representative workloads is presented by Ruffo et al. [44]. First, CBMGs are automatically extracted from Web application log files, and then representative user behavior traces are generated from these CBMGs. Based on these traces, a modified version of the performance testing tool httperf [39] is used to generate the Web traffic. In both approaches, Markov States represent the user interaction with the system. Transitions between these states are annotated with user think times and probabilities.

Zhao and Tian [60] have proven that these models can be used for workload generation. However, one of their limitations is that they are not able to handle the aforementioned inter-request dependencies. In an inter-request dependency an item can only be removed from a shopping cart if items have already been added to that shopping cart. To overcome these limitations, Shams et al. [47] proposed a workload modeling formalism based on Extended Finite State Machines (EFSMs). EFSMs allow a description of valid sequences of user requests within a session. In contrast to the approaches based on Markov Chains, the transitions are labeled with GaAs based on predefined state variables and not with probabilities. Valid sessions are obtained by simulating the EFSMs. Additionally, inter- and intra-session characteristics, such as think times and session length distributions and a workload mix defining the relative frequency of request types, can be specified. Our work combines the modeling approaches based on CBMGs and EFSMs [50]. Thus, probabilistic user behavior modeling is enabled while ensuring that valid sequences of user requests are generated.

Other approaches exist that use analytical formalisms to define workload models. Examples include stochastic form-oriented models [21, 35], probabilistic timed automata [1] or context-based sequential action models [27].

One limitation of the proposed approaches is the need for manual specification of GaAs as they are not extracted automatically from log files. To overcome this challenge, several approaches exist for extracting Behavior Models from system logs in the form of Finite State Machines (FSMs) [10, 41] or EFSMs [56]. We extend the work of Beschastnikh et al. [10] to automatically derive the GaAs for the Application Model, based on temporal invariants mined from the session log files (explained in detail in Sect. 4.4).

### Workload Intensity

An approach focusing on the definition of workload intensities can be found in the work by von Kistowski et al. [49]. Their LIMBO approach allows a DSL-based definition and extraction of variable and dynamic load profiles and workload scenarios over time including seasonal patterns, long-term trends, bursts, and a certain degree of noise. The work proposed by Herbst et al. [25] offers an approach to forecast workload intensities in which suitable forecasting methods are chosen, based on a decision tree and feedback cycles to improve the forecast accuracy. WESSBAS focuses on the specification of the behavior of users and offers basic support for modeling workload intensities (see Sect. 4.3).

### Workload extraction

The extraction of workloads is usually based on request logs [38, 51], design specifications such as UML diagrams [20], or expert knowledge [4].

An approach similar to ours, introducing an abstract intermediate model that defines workload specification independent from the used technology, is defined by Costa et al. [19]. These authors focus on the separation of technology details from the test scenarios. UML diagrams are used as input for creating abstract intermediate models. These model instances are transformed to the load test tools Visual Studio Load Test and HP Loadrunner. As UML diagrams are often not available or not detailed enough, we propose to extract the intermediate language WESSBAS-DSL from log files taking also protocol information and inter-request dependencies into account.

### Workload modeling for performance models

Architecture-level performance models [8, 30, 40] allow the modeling of usage behavior, e.g., based on UML formalisms. These models also allow the specification of the Workload Intensity. The effort to create performance models in a manual way significantly reduces any benefit to be gained. Thus, approaches for the automatic performance model generation have been proposed [11, 15]. These approaches focus on the automatic extraction of the system-specific details of the SUT, like the system components, the relationship between the components, the component allocations, and the resource demands. However, the workload specifications must still be modeled manually, which requires a lot of effort on the part of the performance expert.

To reduce the complexity of generating different kinds of analytical performance models from architecture-level performance models, several intermediate languages such as PUMA [59] or Klaper [18] were introduced. These approaches only focus on model-based performance evaluation and do not support the definition of workload specifications for session-based application systems.

An approach that combines model-based performance testing with load testing for Web-based systems is introduced by Barna et al. [6, 7]. In their approach, the SUT is modeled as a two-layer queuing model. Then, workload mixes and workload intensities are derived from the model under which software and hardware bottlenecks are saturated. Finally, the test cases are derived and executed on the SUT. The model is automatically tuned, based on feedback loops from the SUT. In contrast to WESSBAS, the user behavior is aggregated on transactional level, e.g., a buy transaction, and not on single-user interactions.

## Workload specification

An overview of the workload specification formalism required to understand the paper is given in Sect. 3.1. The WESSBAS-DSL, which is based on this specification, is introduced in Sect. 3.2.

### Workload specification formalism

The approach described in this paper builds on our previous work on generating probabilistic and intensity-varying workloads for session-based systems [46, 50]; particularly, the workload modeling formalism that extends the work by Menascé et al. [38] and Krishnamurthy et al. [32]. This section introduces the concepts needed to support the remainder of this paper.

**Fig. 2:**
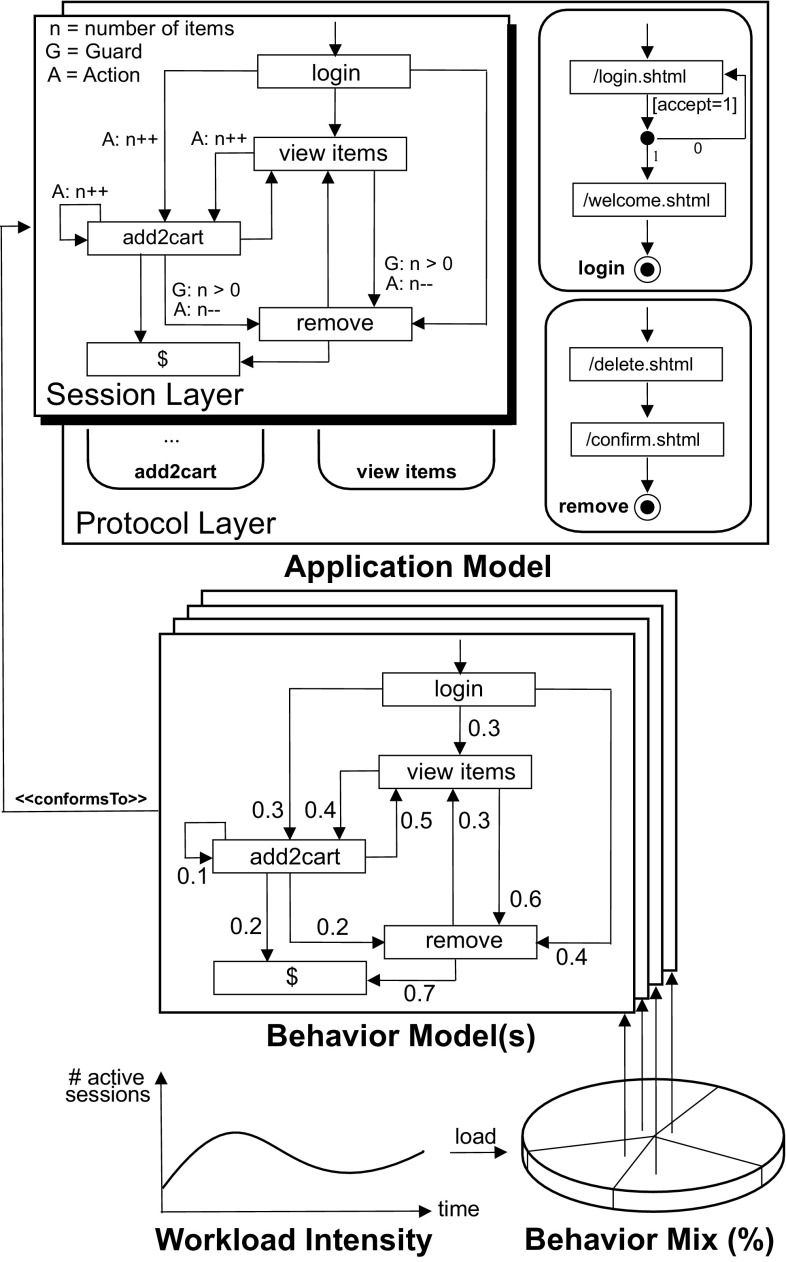
Exemplary workload model (without think times in the Behavior Models)

The workload specification formalism (*Workload Model*) consists of the following components, which are detailed below and illustrated in Fig. 2:

- An *Application Model*, specifying allowed sequences of service invocations and SUT-specific details for generating valid requests.
- A set of *Behavior Models*, each providing a probabilistic representation of user sessions in terms of invoked services and think times between subsequent invocations as Markov Chains.
- A *Behavior Mix*, specified as probabilities for the individual Behavior Models to occur during workload generation.
- A *Workload Intensity* that includes a function which specifies the (possibly varying) number of concurrent users during the workload generation execution.

#### Application model

The Application Model is a two-layered hierarchical EFSM, consisting of a *Session Layer* and a *Protocol Layer*. Inspired by the work of Krishnamurthy et al. [32, 47], the Session Layer is an EFSM in which states refer to system-provided services and allowed transitions among these states/services. These transitions are possibly labeled with GaAs. The EFSM is defined as a 6-tuple [28] $(S\cup \{{\mathtt \$}\}, s_0, V, I, O, T)$ where $S\cup \{{\mathtt \$}\}$ specifies the set of states contained in the Application Model and $s_0\in S$ the initial state; *V* is a finite set of variables; *I* is a set of input symbols; *O* is the set of output symbols; and *T* is a set of possible transitions.

A directed transition $t\in T$ is represented by the 5-tuple $(s_s, i, g_{s,e}, a_{s,e}, s_e)$ in which $s_s$ is the source state of t; *i* is the input where $i\in I$ and *i* may have associated input parameters; $g_{s,e}$ is the guard condition which validates if the transition can be executed according to the current variable values; $a_{s,e}$ defines a function on the variable values called action statement, in case the related application transition fires; and finally, $s_e$ is the target state of *t*.

For each state of the Session Layer, the Protocol Layer contains an associated EFSM, (possibly labeled with GaAs as well) that models the sequence of protocol-level requests to be executed when the Session Layer state is executed.

#### Behavior Models

A Workload Model includes one or more Behavior Models. Each Behavior Model defines probabilistic behavior and think times. Furthermore, each Behavior Model is specified as a Markov Chain and roughly corresponds to the CBMGs introduced by Menascé et al. [38]. Each Behavior Model $$\mathcal{B}$$ is defined as the tuple $$(MS\cup \{{\mathtt \$}\}, ms_0, P, $$ $$TT, f_{tt}, BT)$$. *MS* specifies the set of Markov States contained in the Behavior Model with initial Markov State $$ms_0\in MS$$ and exit state $$\mathtt {\$}$$. Each Markov State is associated with exactly one Application State of the Application Model. $$P = [p_{s,e}]$$ is a $$n\times n$$-matrix of transition probabilities, with $$n = |MS\cup \{{\mathtt \$\}}|$$. Think times are specified as an $$n\times n$$-matrix $$TT = [tt_{s,e}]$$, with $$n = |MS\cup \{{\mathtt \$\}}|$$. The distribution function $$f_{tt}$$ specifies the probability distribution of the think times. For instance, the think times may be specified using a Gaussian distribution. *BT* is a set of transitions in the Behavior Model.

A transition in a Behavior Model $bt\in BT$ is represented by the 4-tuple $(ms_s, p_{s,e}, tt_{s,e}, ms_e)$ in which $ms_s$ is the source Markov State of *bt*. A matrix entry $p_{s,e}\in P$ defines the probability for a transition from Markov State $ms_s$ to Markov State $ms_e$. When the probability of $p_{s,e}=0$, then the transition cannot be executed. A matrix entry $tt_{s,e}\in TT$ defines the think time for a transition from state $ms_s$ to state $ms_e$. Finally $ms_e$ is the end Markov State of the transition *bt*.

The Behavior Models can also be defined as *absolute* Behavior Models $\mathcal{AB}$ represented as the tuple $(MS\cup \{{\mathtt \$}\}, ms_0, A, STT, BT, f_{tt})$. Then, the matrix $A = [a_{s,e}]$ specifies a $n\times n$-matrix of absolute transition counts. Furthermore, the matrix $STT = [stt_{s,e}]$ represents a $n\times n$-matrix of accumulated think times of the transitions.

The advantage of separating the Application Model and the Behavior Models is that the Protocol Layer and the GaAs for the transitions of the Session Layer have to be specified only once. Otherwise, this information would need to be added to each Behavior Model.

#### Behavior Mix

The Behavior Mix is a set $\{(\mathcal{B}_{0}, r_0), \ldots , (\mathcal{B}_{m-1}, r_{m-1}) \}$, which assigns a relative frequency $r_i$ to the Behavior Model $\mathcal{B}_{i}$. A tuple $(\mathcal{B}_{i}, r_i)$ indicates that sessions which correspond to the Behavior Model $\mathcal{B}_{i}$ are generated with a relative frequency of $r_i\in [0, 1]$. That is, each $r_i$ denotes a probability value and the sum of all values must be 1, corresponding to 100 %.

#### Workload intensity

The Workload Intensity for an experiment is specified in terms of the number of active sessions, i.e., the number of virtual users being simulated concurrently. A generated session is considered active, while the workload generator submits requests based on the corresponding probabilistic session model (the exit state of the Behavior Model has not been reached). A function $n : \mathbb {R}_{\ge 0} \rightarrow \mathbb {N}$ specifies this number *n*(*t*) of active sessions relative to the elapsed experiment time *t*. Particularly, this allows for generating a varying workload intensity profile, e.g., based on measured workload data.

#### Workload generation process

During the workload generation process for a SUT, the model is used as follows (see Fig. 2):

The Workload Intensity specifies the number of active sessions. For each newly created session, the Behavior Mix determines the user type to be emulated next by selecting the corresponding Behavior Model $\mathcal{B}_{i}$ based on the assigned relative frequencies $r_i$. In the selected Behavior Model, a probabilistic sequence of services is generated according to the transition probabilities specified in the related Markov Chain. Furthermore, the GaAs of the Session Layer are taken into account in order to generate valid sequences.

**Fig. 3:**
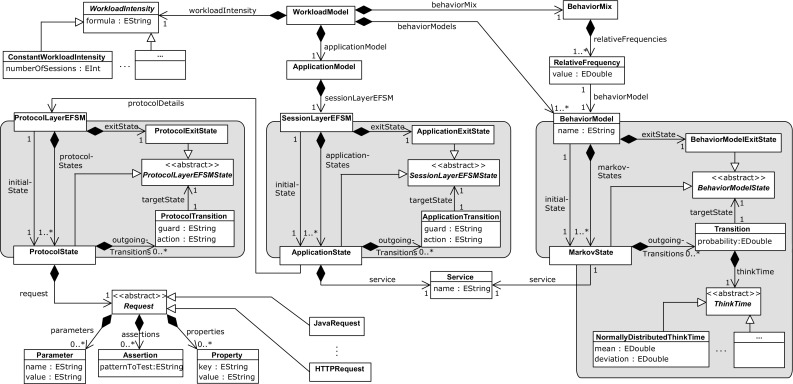
WESSBAS-DSL classes and relationships

Assume that the Behavior Model $\mathcal{B}_{i}$ is currently in the Markov State $view\_items$ ($view\_items \in MS$) and the current variable *n* ($n \in V$) has the value one. First, based on the transitions *T* modeled in the Session Layer from application state $view\_items$ ($view\_items \in S$) to the following states, it is validated which guard conditions are satisfied—in the example, the transition from $view\_items$ to *add*2*Cart* and *remove*. As the number of items *n* is one, the guard $G: n > 0$ to transition *remove* is true. The transition to *add*2*Cart* has no guard and can therefore always be executed. Second, based on the probabilities specified in the matrix *P*, the next transition is chosen—40 % of cases to *add*2*Cart* and 60 % to *remove*. Third, the action(s) on the variable value(s) will be executed. When *remove* is chosen, the value of *n* is decreased by one; when *add*2*Cart* is chosen, the value is increased by one. Finally, the think time is taken from the think time matrix *TT* for this transition. After the think time has elapsed, the Behavior Model moves to the next state and the service is executed according to the specified EFSM of the Protocol Layer.

### WESSBAS-DSL

The WESSBAS-DSL follows the workload modeling formalism introduced in the previous section and denotes a language for expressing such models. In our approach, the WESSBAS-DSL is used as an intermediate language between the construction of SUT-specific but tool-agnostic workload models on the one side, and the generation of corresponding inputs to load testing tools and performance models on the other side. WESSBAS is implemented as an Ecore-based meta-model using the benefits and tool support of the Eclipse Modeling Framework (EMF) [48]. The meta-model is enriched with constraints (specified in the common Object Constraint Language OCL), for checking the validity of model instances. The DSL structure offers a high degree of flexibility and extensibility. The remainder of this section introduces the core concepts.

An overview of the WESSBAS-DSL classes and relationships as a language for the introduced workload specification is presented in Fig. 3. The parent class Workload Model consists of the Application Model, the Workload Intensity, the Behavior Mix, and Behavior Models.

The representation of the Application Model corresponds to the two-layered structure of that component, including EFSMs for both the Session Layer and the Protocol Layer. States of the Session Layer EFSM, shortly referred to as Application States, are associated with services and with a Protocol Layer EFSM. Services are use cases, e.g., signing on to a system or adding an item to the shopping cart (see Fig. 2). The states of the Protocol Layer EFSM are associated with specific requests, which might be of type HTTP, Java, BeanShell, SOAP, etc.; the set of currently supported request types can be extended easily by deriving additional subclasses from the common base class. Mention should be made of the difference between properties and parameters of a request: Properties correspond to the information that is required for sending a request (e.g., domain, path, or port number of a targeted server); parameters denote values to be sent with the request (e.g., input data for a Web form). The transitions in the Session Layer and Protocol Layer EFSMs can be labeled with GaAs. An example can be seen in Fig. 2. The user action *remove* can only be called when the number of items is greater than zero.

Behavior Models are modeled as Markov Chain, with each (Markov) State also being associated with one service. Thus, each Markov State is assigned exactly to one Application State of the Session Layer. Transitions of the Behavior Models are labeled with probabilities and think times. Currently supported think times are of type Gaussian, that is, they follow a normal distribution, indicating mean and (standard) deviation values as parameters. Other think time implementations can be integrated easily by using the abstract class *ThinkTime*. Exit states are modeled explicitly and are—in contrast to Markov States—not associated with services as they do not provide a service to the user. Each Behavior Model is associated with a relative frequency to define the Behavior Mix, and it is stored as a double value in a dedicated class. These frequencies are contained in the Behavior Mix. The Workload Intensity is stored as a string attribute in the dedicated class *WorkloadIntensity* that also serves as a base class for all types of workload intensities. The Workload Intensity can be specified as a formula to define varying workloads or as a fixed number for constant workloads.

**Fig. 4:**
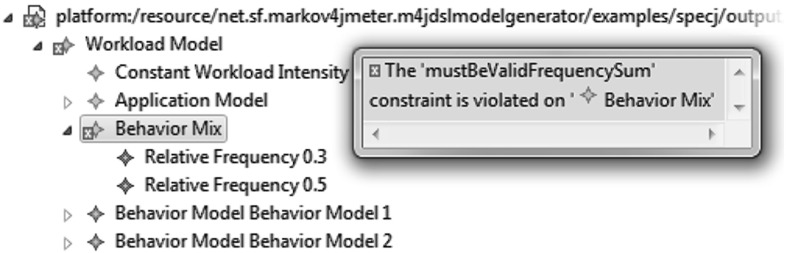
Example of a WESSBAS-DSL model with a violated constraint (no Behavior Mix frequency sum of 1.0), opened in an EMF Form Editor

Even though the WESSBAS-DSL is independent of specific performance evaluation tools, it includes all core information required for generating workload specifications that build on the described workload modeling formalism. The implementation of the WESSBAS-DSL as an Ecore meta-model offers the benefits of EMF tools such as EMF Form Editors or serialization support. In particular, WESSBAS-DSL instances can be viewed, validated, and modified in an editor, before being passed as input to any transformation process. For the validation, OCL constraints have been defined with the use of EMF *OCLinEcore* tools. An example of a violated constraint is shown in Fig. 4.

These constraints ensure that, for example, attributes such as probabilities are valid, state names are unique, and transitions of Behavior Models correspond to those of the Session Layer EFSM. An overview of all implemented OCL constraints is given in [45].

## Extracting WESSBAS-DSL instances

As the manual creation of workload models requires much effort, this section presents the process for extracting WESSBAS-DSL instances automatically based on recorded system logs.

The remainder of this section details the six-step procedure to obtain a WESSBAS-DSL instance, comprising (i) the extraction of a session log from the production system (Sect. 4.1), (ii) the clustering-based extraction of the Behavior Mix (Sect. 4.2), (iii) the extraction of the Workload Intensity (Sect. 4.3), (iv) learning of GaAs (Sect. 4.4), (v) calculation of conditional probabilities (Sect. 4.5), and (vi) the generation of a complete WESSBAS-DSL instance from the Behavior Mix (Sect. 4.6).

### Monitoring and session log generation

The extraction of WESSBAS-DSL instances is based on a so-called *session log* obtained from raw session information, recorded from a running application system. Raw session information is usually provided by *request logs* generated by monitoring facilities, comprising the associated requests to system-provided services with a session identifier and timestamps for the request and completion time. A typical example is the HTTP request log provided by common web servers [38], or tracing information obtained from application-level monitoring tools [52].

**Fig. 5:**
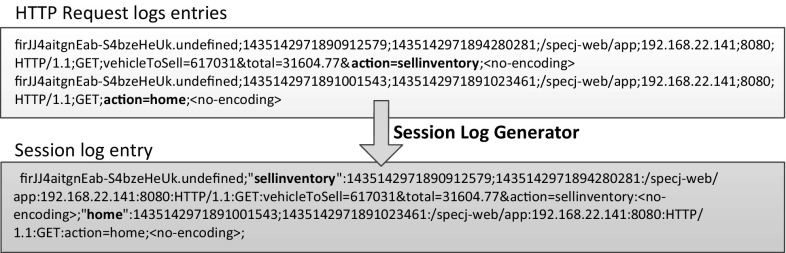
Example HTTP log (recorded with Kieker) and resulting session log

For each HTTP request, the following information is mandatory to create WESSBAS-DSL instances (example see Fig. 5): “session identifier”, “request start time”, “request end time”, and “request URL”. In order to further create the Protocol Layer for the generation of executable load tests, the following request information is required as well: “host IP”, “port”,“method” (GET/POST), “protocol”, “parameter with parameter values”, and “encoding”. These Protocol Layer information is not required for the creation of PCM models.

The request information will be transformed to a session log, which can be processed in the next step by the Behavior Mix Extractor. During the transformation, the requests are grouped by the specified session identifier (e.g., session identifier, client IP, or user ID), giving access to the sequence and timing information of subsequent service requests within a session (see Fig. 5). In each line, the leading number denotes a unique session identifier followed by the sequence of extracted services. A service execution is identified by its assigned name in quotes followed by its start time and end time, and the protocol information.

A service defines a specific user interaction with the system, like clicking a link on a Web page. Each service will later be translated to a service of the WESSBAS-DSL (see Fig. 3). Thus, the services also represent the states in the Session Layer and the Markov States of the Behavior Models. As the identification of the services is dependent on the respective application, its translation must be specified manually. The user can specify that each distinct requested URL is a service. However, there are applications where different URLs represent the same service. In other applications, the same base URL is used and the services can only be distinguished based on submitted parameters. The translation can be defined using the URLs, the parameter names, or corresponding parameter values of the request. For instance, a HTTP request parameter called *action* has the values *sellinventory* or *home* (see Fig. 5); the values of this parameter can then be used to distinguish the two services.

As each monitoring facility can generate different log formats (e.g., different delimiters or date and time formats), the WESSBAS approach provides tool support for transforming the raw logs to the session log, named the Session Log Generator. The tool enables the user to specify the input raw logs files and then to manually define how the required session data are extracted from the raw logs to the session log. Our approach is independent from specific monitoring solutions, so using the Session Log Generator is advantageous. Furthermore, these rules must only be configured once and can then be reused each time new session logs in the same format are available.

**Fig. 6:**
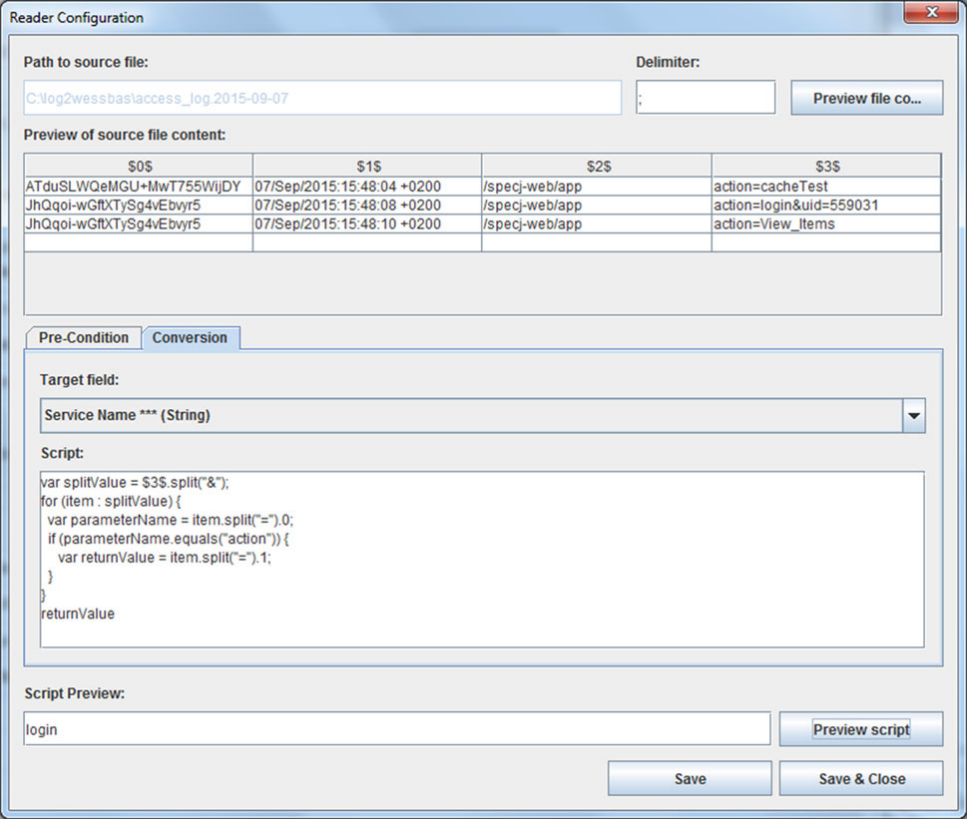
Exemplary translation rule in the Session Log Generator

With this tool, the translation of the request data to the service names can be defined. We integrated the Java Expression Language (JEXL)^3^ to enable the user to define these translation rules. An example of how to use the Session Log Generator to define a translation rule can be found in Fig. 6. A HTTP access log is read in by the Session Log Generator. This log consists of session identifiers, time stamps, request URLs, and request parameters for each request. To specify the service name of the resulting session log, the value of the parameter “action” will be identified. For the second row of the log, the value “login” is identified.

As users can exit their sessions at any time or can have long inactivity periods, the determination of service requests belonging to a session is required as well. The determination depends on the available session identifier within the raw logs. In case an identifier generated by a Web server is used, no further information has to be specified. Because a session time out is configured in each web server, users with a long inactivity period are automatically assigned to a new session identifier. If client IP addresses or user IDs are used, a threshold for the maximum allowed time between two user requests can be specified [38]. In case this threshold is exceeded, the current sequence of service requests is split. Then, the following requests are considered to belong to a new session with a unique session identifier. This threshold can also be defined in the Session Log Generator.

### Clustering-based Behavior Mix extraction

The Behavior Mix Extractor extracts the Behavior Mix and the corresponding Behavior Models based on the created session log. The Behavior Mix is determined by identifying different groups of customers with similar navigational patterns. As proposed by Menascé et al. [38], clustering methods can be used to support this task. The identification of different customer groups has several advantages. For example, the system can be optimized upon these navigational patterns. Furthermore, the impact of different Behavior Mixes on the performance can be evaluated, e.g., investigating the performance impact of an increased fraction of a customer group. Lastly, the goal of the clustering is to obtain a relatively small number of clusters to reduce the complexity and to increase the comprehensibility of the resulting Behavior Mix.

In this paper, we focus on clustering with the centroid-based X-means algorithm, which is an improved version of the well-known K-means algorithm [42]. The advantage of X-means over K-means is that it is not mandatory to specify the number of clusters K in advance by the user. The user provides a minimum and a maximum number of resulting clusters, and the algorithm determines how many clusters are best suited. The evaluation of K-means clustering is very costly because the results of the K-means must repeatedly be evaluated with different numbers of K [9]. Furthermore, the X-means algorithm scales better and the risk of finding local minima is lower. The X-means clustering algorithm is integrated into our proposed approach using the data mining framework Weka [24]. Other algorithms can be integrated accordingly.

The Behavior Mix Extractor reads the session log file and first transforms each session entry into an *absolute* Behavior Model. This model is composed of a $n\times n$-matrix *A* defining the transition counts and of a $n\times n$-matrix *STT* defining the accumulated think times. We remind that we use the workload specification introduced in Sect. 3.1.2. Because Weka cannot handle matrices as clustering input, each matrix *A* of each *absolute* Behavior Model is transformed into a vector $V = v_1,...,v_n$ by mapping each value $a_{s,e}$ to a value of the vector by:

1 $$\begin{aligned} a_{s,e} \rightarrow v_{(e+((s-1) \cdot n))} \end{aligned}$$

Think times are not part of the clustering as they have no impact on the navigational patterns. As future work, it could be of interest that the Behavior Models are also clustered using the think times. During the clustering in the first step, a central vector $V'$, called centroid, is determined randomly for each cluster. Each centroid represents a cluster and is the mean of the instances (in our case, sessions represented as transition counts matrices of the *absolute* Behavior Models) $AB_m = [A_m], m = 1,...,M$ in that cluster. Then, the clustering algorithm iterates several times over the dataset and assigns instances to the nearest cluster centroid until no instance no longer changes the cluster. After each iteration, each centroid $V'$ is recalculated by:

2 $$\begin{aligned} v'_{i} = \frac{\sum \nolimits _{i=1}^m v_i}{m} \end{aligned}$$

The distance between the instances is calculated using the Euclidean distance metric. During the calculation of a distance, the attributes of the instances (represented as a vector) can be normalized to a value between zero and one. Without data normalization, the attributes with the highest variance drive the clustering. That means, in our case, high transition counts have a high influence on the clustering. In order to figure out the best settings, both the normalized and the non-normalized Euclidean distances will be evaluated in Sect. 7.4.1. Other distance metrics like Manhattan distance or Chebyshev distance could be used as well.

The *relative* Behavior Models are calculated as proposed by Menascé et al. [38]. First, each centroid vector $V'$ is transformed back to a matrix $A'$. Then the corresponding think time matrix $STT'$ is calculated by accumulating the think times of the single *absolute* Behavior Model instances $AB_m$ within each cluster:

3 $$\begin{aligned} stt'_{s,e} = \sum _{i=1}^m stt_{i_{s,e}} \end{aligned}$$

As a result, the centroids represent the *absolute* Behavior Model of the corresponding cluster. Afterward, these absolute transition count matrices $A'$ are transformed to relative $n\times n$-matrices *P*, defining the transition probabilities. Furthermore, the matrix $STT'$ will be transformed to the matrix *TT* representing the mean think time per transition.

4 $$\begin{aligned} p_{s,e}= & {} \frac{a'_{s,e}}{\sum \nolimits _{i=1}^n a'_{s,i}} \end{aligned}$$

5 $$\begin{aligned} tt_{s,e}= & {} \frac{stt'_{s,e}}{a'_{s,e}} \end{aligned}$$

Finally, the relative frequency *r* of each Behavior Model is calculated by dividing the number of instances *m* within each cluster by the overall number of session instances in the session log.

### Workload Intensity extraction

The Workload Intensity is automatically analyzed based on the session log and included into the resulting model. The maximum and the average number of concurrent sessions is determined. The user can configure which of these values should be included into the WESSBAS instance. During test execution, this number represents the number of concurrent threads, each starting a new session after the previous session is finished [5]. Testing the SUT with peak or average loads is sufficient for many application systems.

In order to be able to integrate varying load intensities, for example to test dynamic resource allocations used in virtualized data centers and cloud computing, the LIMBO approach proposed by von Kistowski et al. [49] could be integrated in WESSBAS. The LIMBO approach generates a load intensity model from log files describing the session arrival rate over time using mathematical functions. This meta-model is also implemented using EMF tools and can be combined with the WESSBAS-DSL Workload Intensity definition. Furthermore, there are already available extensions for JMeter^4^ and PCM [34]

### Automatic learning of guards and actions

As stated in the previous section, transitions of the Application Layer are optionally labeled with GaAs. As workload specifications might generate invalid paths using solely probabilistic transitions, an important task to be considered is the identification of GaAs. This leads to the fact that errors might occur or that less demand is generated on the system resources during load testing, as the user behavior is incorrectly represented. The generated load on the system could be incorrect, and performance characteristics, such as CPU utilization or response times, might be different than using correct user behavior [47].

**Fig. 7:**
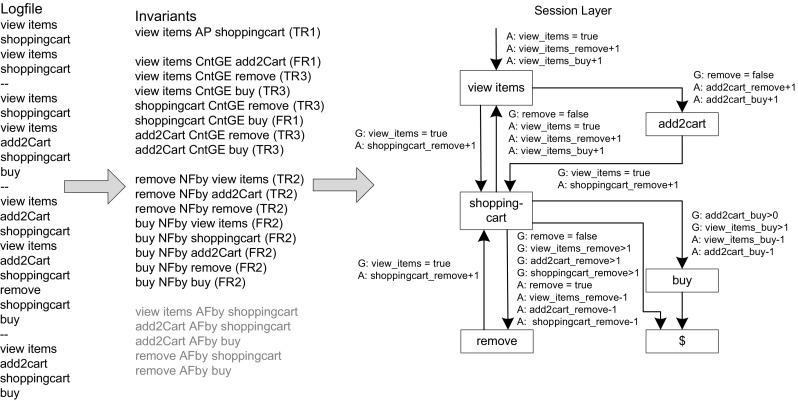
Exemplary translation of temporal invariants to GaAs

We build on the approach introduced by Beschastnikh et al. [10], called Synoptic, to learn the GaAs automatically. Beschastnikh et al. define three different kinds of so-called temporal invariants representing relationships between event types (in our case service invocations) that are true over all input traces. Their approach was easily integrated into our WESSBAS framework as the temporal invariants can also be extracted from the same session log file. Three different kinds of invariants are defined and extracted [10]:

- *a* Always Followed by *b* (AFby): Whenever the event type *a* appears, the event type *b* always appears later in the same session trace.
- *a* Never Followed by *b* (NFby): Whenever the event type *a* appears, the event type *b* never appears later in the same session trace.
- *a* Always Precedes *b* (AP): Whenever the event type *b* appears, the event type *a* always appears before *b* in the same session trace.

In our approach, we need to identify guards which must be true to execute the associated application transition. This identification is important in order to generate valid user navigations as some user actions can only be executed after other specific user actions have been executed.

We are not only interested in the sequence of user actions, but it is also important to specify how often an user action is executed before another. For instance, the removal of items in a shopping cart is dependent on the number of items added previously to the shopping cart. The user can only execute the user action *remove* as often as there are items in the shopping cart. Therefore, we introduce a new type of temporal invariant, which is a subset of the *AP* invariant:

- Count *a* Greater or Equal as Count *b* (CntGE): For each *AP* invariant the number of executions of *a* is always greater or equal compared to the number of executions of *b* in the same session trace. Additionally, the minimal difference between the execution of *a* and *b* is determined.

Figure 7 illustrates a simple example of how the temporal invariants are translated to GaAs. The simple logfile contains four sequences of user actions, each representing a session. From these sessions, 21 temporal invariants are extracted and translated to GaAs of the Session Layer.

Of the four invariant types, *AFby* cannot be used. The condition *AFby* does not mean that *a* must always be executed before *b* as seen in the example (see Fig. 7). The invariant *add2Cart AFby shoppingcart* cannot be used as guard as the user action *shoppingcart* can also be called from the state *view items*. Thus, the user action *add2cart* is not a prerequisite to executing the user action shoppingcart.

Not all of the remaining temporal invariants are required to generate valid user behavior. Furthermore, the translation of all resulting invariants into GaAs would make the workload specification quite complex. Therefore, we use the following filter rules to check whether each temporal invariant is required:

- Filter rule 1 (FR1): Assuming a temporal invariant *a* to *b* exists. If state *a* and state *b* are directly connected and state *b* has only one incoming transition from state *a* and the minimal difference is zero, then the guard is not required. In this case state *a* is always called before state *b*. Furthermore, state *b* cannot be called more frequently than state *a*. For instance, *shoppingcart CntEG remove* does not need to be considered, as *remove* can only be called when *shoppingcart* is called.
- Filter rule 2 (FR2): A path from *a* to *b* exists. This filter rule is important for the NFby invariant, as Synoptic does not check whether *a* can be followed by *b*.

For the remaining invariants, we define the following translation rules. The invariants *NFby* and *AP* are translated into Boolean state variables, whereas the new invariant *CntGE* is translated to a numeric state variable.

- Translation rule 1 (TR1): Boolean variable *a* for *AP*
  - Action: If event *a* is executed, the variable *a* is set to true.
  - Guard: Each transition to event *b* validates if *a* is true.
- Translation rule 2 (TR2): Boolean variable *a* for *NFby*
  - Action: If event *a* is executed, the variable *a* is set to true.
  - Guard: If a path from *a* to *b* exists, then each transition to event *b* checks if *a* is false.
- Translation rule 3 (TR3): Numeric variable *a_b* for *CntGE*
  - Action: If event *a* is executed, the value of a variable called *a_b* is increased by one.
  - Guard: Each transition to event *b* first checks whether variable *a_b* is greater than the minimal difference between the execution of *a* and *b*. If yes and the transition is executed, *a_b* is decreased by one.

In our example we identified nine relevant invariants which are translated into GaAs. The precondition for a transition to be executed is that all guards must be true (logical conjunction). A good example is the temporal invariant *add2Cart CntGE remove*. The user action *remove* can only be called when the user has previously added an item to the shopping cart. Thus, the transition from *shoppingcart* to *remove* is only executable when the condition *add2Cart_remove* is greater than one. In this case, the condition must be greater than one, as the minimal difference of request counts between *add2cart* and *remove* is one. We represent the guard as a numeric variable for this type of relationship. When the previously required user action *add2Cart* is executed, the variable *add2Cart_remove* is increased by one. Later, the transition from *shoppingcart* to *remove* validates whether this condition is true or not. If this condition is true, the transition is fired and *add2Cart_remove* is decreased by one.

### Calculation of conditional probabilities

Because we combine GaAs with probabilities, the calculation of conditional probabilities may be required. The conditional probability is the probability that a transition will be executed given that the corresponding guard condition is true. The conditional probability can be considerably different from the probability $p_{s,e}$ (see Sect. 3.1.2) that we have extracted in the Behavior Mix Extractor for each Behavior Model (see Sect. 4.2).

**Fig. 8:**
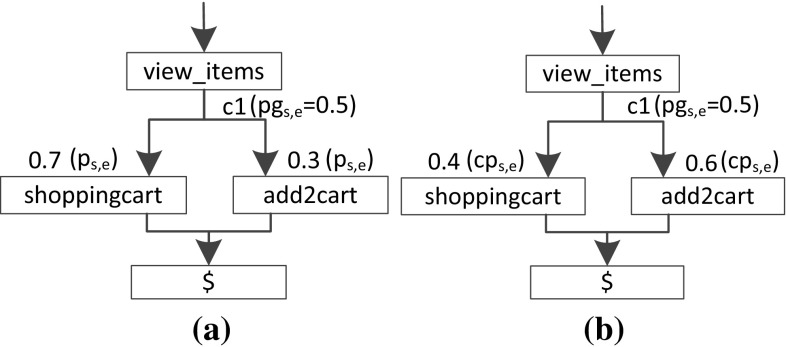
An exemplary Behavior Model with measured probabilities and with conditional probabilities. **a** Measured probabilities. **b** Conditional probabilities

To exemplify this, assume we have extracted a simple Behavior Model from a session log (see Fig. 8a). This model consists of a transition from *view_items* to *add2cart* that is executed in 30 % of cases and of a transition from *view_items* to *shoppingcart* executed in 70 % of cases. We assume that the probability that the guard condition *c*1 is true is 50 %. When we execute or simulate this Behavior Model, the transition from *view_items* to *add2cart* would be executed in only 15 % (i.e., 50 % $\cdot$ 30 %) of cases and the transition from *view_items* to *shoppingcart* in 85 % of cases. As this result is different from the initially measured transition probabilities, the request counts of the extracted workload specification would be different from the request count of the original workload. In the remainder of this section, we propose a heuristic to calculate the conditional probabilities. As future work, we will examine other approaches like Bayesian networks as well.

In the first step, in order to calculate the conditional probability, we have to obtain the probability for each transition that the respective guard condition is true. Let $pg_{s,e}$ be the probability that the guard *g* from state $s_s$ to state $s_e$ is true.

Based on the session log we can calculate this value by computing the relative frequency of each transition that the corresponding guard conditions is true: From the measured session log we take the sessions belonging to a Behavior Model $\mathcal{B}$, as obtained by the clustering. Then, we interpret each session by iterating the transitions according to the measured state sequence. Within each state, we determine the potential transitions to the next states, according to the Behavior Model. Afterward, for each transition the value of the guard condition identified in the previous step (see Sect. 4.4) is determined. Then, the next state is chosen according to the state sequence and the corresponding action is executed. This way, we calculate the value of $pg_{s,e}$ for each transition by:

6 $$\begin{aligned} pg_{s,e} = \frac{ Count g_{s,e} is true }{ Count g_{s,e} is evaluated } \end{aligned}$$

For example, we have 100 sessions for the exemplary Behavior Model of Fig. 8. Within these sessions, the state *view_items* occurs 100 times. Each time the state *view_items* is examined, we evaluate the guard conditions of the potential transitions to *add2cart* and to *shoppingcart*. Assume that in 50 cases the condition of the transition to *add2cart* was true and in 100 cases the condition of the transition to *shoppingcart* was true. Then, *pg* for the transition to *add2cart* is 50 % (i.e., 50/100) and to *shoppingcart* it is 100 % (i.e., 100/100).

In the second step, we calculate the conditional probabilities of all transitions where the probability that the guard condition is smaller than one, as in these cases the probability must be increased. For each transition *bt* of a Behavior Model $\mathcal{B}$ we calculate the transition probability $cp_{s,e}$ under the condition that the corresponding guard is true according to Kolmogorov [29] by:

7 $$\begin{aligned} cp_{s,e} = \frac{p_{s,e}}{pg_{s,e}}, \quad \forall \{ { bt} \mid 0< pg_{s,e} < 1 \} \end{aligned}$$

In our example, the conditional probability $cp_{s,e}$ for the transition *view_items* to *add2cart* would be adjusted to 60 % (i.e., 30 %/50 %) as $0<pg_{s,e} <1$ (see Fig. 8b).

In the third step, to ensure that the sum of the conditional probabilities from one state to the following states is again 100 %, we have to adjust the probabilities of the transitions where $pg_{s,e}$ = 1. All transitions from state *s* to the following states $E = e_1,...,e_N$ are identified and adjusted by:

8 $$\begin{aligned} cp_{s,e} = p_{s,e} \cdot \frac{1-\sum \nolimits _{i=1}^n cp_{s,e_i}}{\sum \nolimits _{i=1}^n p_{s,e_i}}, \quad \forall \{ { bt} \mid pg_{s,e} = 1 \} \end{aligned}$$

In our example, the percentage of transitions from *view_items* to *shoppingcart* is 70 % and the conditional probability of *view_items* to *add2cart* is 60 % (see previous step). Therefore, we adjust the probability to 40 % (i.e., 70 % $\cdot$ (100–60 %)/(70 %)).

If all guard conditions $pg_{s,e}$ from one state to the following states are smaller than one, we have to adjust the probabilities by:

9 $$\begin{aligned} cp_{s,e} = cp_{s,e} \cdot \frac{1}{\sum \nolimits _{i=1}^n {cp_{s,e_i}}} \end{aligned}$$

The originally calculated probabilities within the Behavior Models are adjusted according to the conditional probabilities. Thus, during the transformation to performance evaluation tools only the calculated conditional probabilities are taken into account.

### Generating WESSBAS-DSL Instances

The next task is to transform the extracted Behavior Models, the Behavior Mix, the Workload Intensity, and the GaAs to a valid WESSBAS-DSL instance, which can be further transformed to load generation tools and performance models. Therefore, the *WESSBAS-DSL Model Generator* (Fig. 1) performs the following steps automatically:

1. Construction of an Application Model, based on SUT-specific states and transitions,
2. integration of the Behavior Mix including the extracted Behavior Models,
3. integration of the Workload Intensity definition,
4. integration of Guards and Actions and conditional probabilities, and
5. extraction and integration of input parameters.

The WESSBAS-DSL Model Generator reads the resulting Behavior Models, builds a corresponding Session Layer EFSM, and assigns a Protocol Layer EFSM to each Application State. The transitions of the Session Layer EFSM are set according to the Behavior Models. A transition from service *a* to service *b* is set, when in one of the Behavior Models a corresponding transition with probability greater than zero exists. From each service, a transition to the final state is set, as each session can be canceled by the user at any time.

The structure of our Protocol Layer EFSMs has one Protocol State per EFSM, providing exactly one request being sent in an Application State. A DSL that allows the definition of more complex, protocol-specific EFSMs, e.g., failed user logins, denotes a future work issue. In our case, we extract HTTP requests from the SUT. For other request types, e.g., Java requests, further extensions need to be developed. For each Protocol State, we integrate the information required to create executable load tests (see Sect. 4.1), like the “host IP” and the “port”, and add this information as property to the request type of the Protocol State. We also integrate the used parameters with the associated parameter values. For each parameter of a Protocol State, all parameter values are stored as a list and can later be reused by load test generators.

The integration of Behavior Mix and Behavior Models includes the construction of corresponding WESSBAS-DSL elements (see Fig. 3). Each Behavior Model is created based on the Behavior Models extracted in the previous step. Each available service is mapped exactly to a Markov State. Finally, the transitions are created for all transitions within the Behavior Models with probability greater than zero.

## Generating JMeter test plans

A given WESSBAS-DSL instance can be transformed into a corresponding JMeter Test Plan. We developed a publicly available extension, called Markov4JMeter [50], for the well-known load generator Apache JMeter, which allows us to define and execute these workload specifications. JMeter supports the generation of workloads of various types of systems, not limited to Web-based systems.

**Table 1:** Probabilities and think times of a Behavior Model (see Fig. 2)

	Login*	View items	Add2cart	Remove	$
Login*	0.0; n(0 0)	0.3; n(3 0.3)	0.3; n(3 0.2)	0.4; n(5 0.9)	0.0; n(0 0)
View items	0.0; n(0 0)	0.0; n(0 0)	0.4; n(4 0.4)	0.6; n(2 0.8)	0.0; n(0 0)
Add2cart	0.0; n(0 0)	0.5; n(5 0.8)	0.1; n(4 0.1)	0.2; n(4 0.2)	0.2; n(7 0.9)
Remove	0.0; n(0 0)	0.3; n(2 0.5)	0.0; n(0 0)	0.0; n(0 0)	0.7; n(5 1.0)

* Initial Markov State of a Behavior Model

**Table 2:** Mapping of WESSBAS-DSL concepts to (Markov4)JMeter elements

WESSBAS-DSL	Markov4JMeter elements
Session Layer FSM	Markov States (+outgoing transitions)
Protocol Layer FSMs	JMeter Elements (Markov State children)
Workload Intensity	MSC (Session Arrival Controller)
Behavior Models	MSC (frequency table) $\rightarrow$ CSV-files
Behavior Mix	MSC (frequency table)
Input parameter	User defined variables
Guards and actions	User parameters

*MSC* Markov Session Controller

The *Test Plan Generator* (Fig. 1) reads a serialized WESSBAS-DSL instance, as described in Sect. 4.6, from file and constructs a further XMI structure, which can be processed by the JMeter tool. The XMI output is generated via the JMeter API and denotes a JMeter-typical tree structure of Test Plan elements, including Markov4JMeter-specific elements, namely *Markov States* and a *Markov Session Controller*, which are provided by the Markov4JMeter add-on for JMeter [50].

The probabilities and think times of the Behavior Models are defined in external comma-separated value CSV files. These CSV files are read by the Markov-4JMeter extension and consist of the transition probabilities and the think times between the Markov States represented as a matrix (see Table 1). For instance, the probability of the transition *add2cart* to *remove* is 20 % with a mean think time of 4 s and a standard deviation of 0.2 s. As the normal distributed think times can be below zero, Markov4JMeter automatically handles negative values as zero. These files were also automatically created by the JMeter Test Plan Generator.

**Fig. 9:**
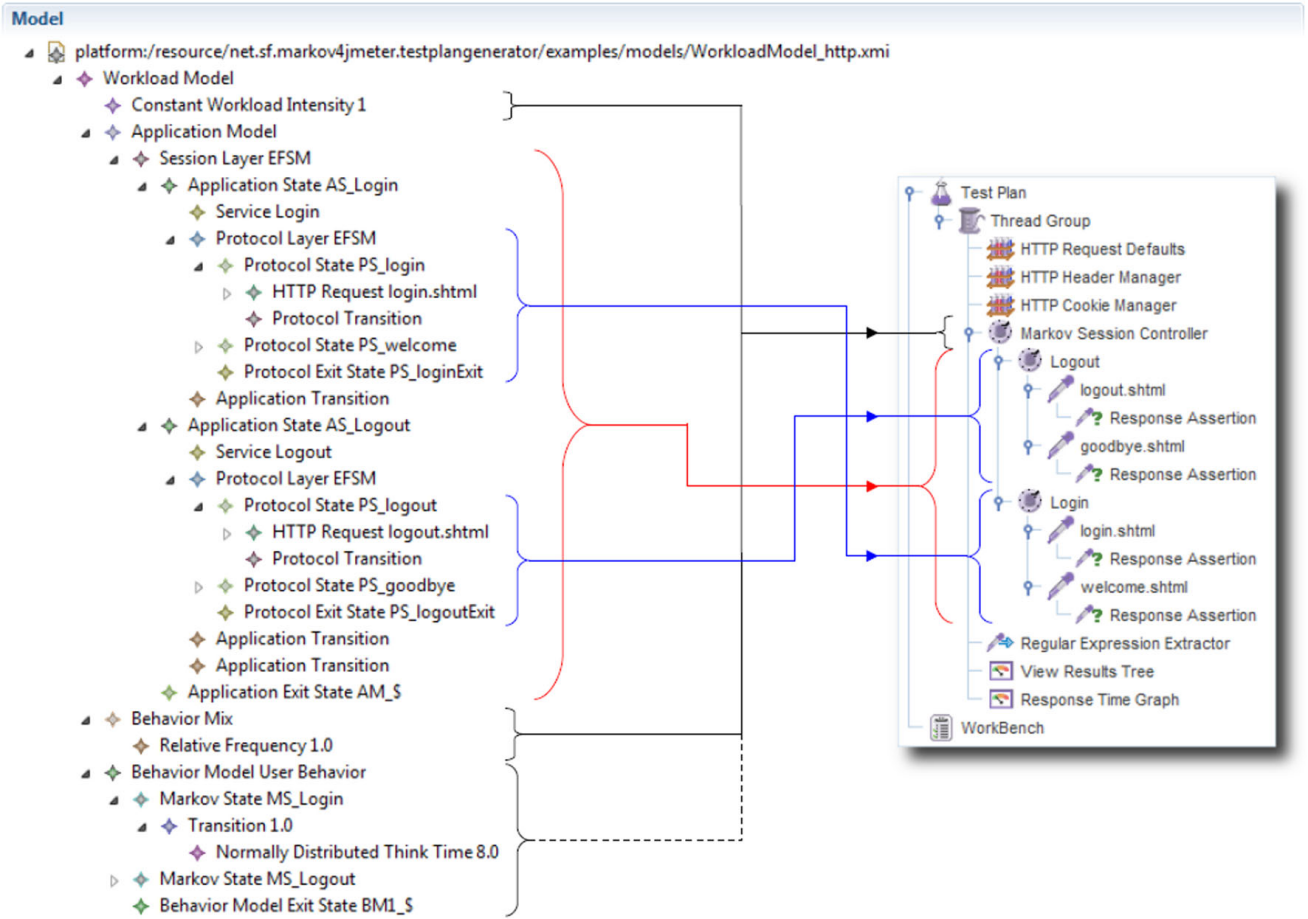
Example mapping of WESSBAS-DSL instances to (Markov4)JMeter Test Plan elements

On start of the transformation process, WESSBAS-DSL input models are validated with respect to the OCL constraints discussed in Sect. 3.2. The core transformation process builds on a mapping between WESSBAS-DSL concepts and (Markov4)JMeter Test Plan elements. An overview of the underlying mappings is given in Table 2.

A Session Layer EFSM in the WESSBAS-DSL is mapped to a corresponding set of Markov States in JMeter. Each Markov State includes its individual set of outgoing transitions with GaAs, for defining the validity of state execution sequences. For each guard and action parameter, a so-called User Parameter is created. In contrast to User Defined Variables, User Parameters are specific for each thread. User Parameter and User Defined Variables are Test Plan elements which are provided by JMeter. The name of a Markov State in the resulting JMeter Test Plan corresponds to the name of the state’s associated service in the WESSBAS-DSL instance. Protocol Layer FSMs are modeled as child elements of Markov States in the tree-structured result and they are constructed with the use of JMeter controllers and samplers according to their related WESSBAS-DSL structure.

The values of each request parameter are created as a User Defined Variable with the parameter name and a list of the measured parameter values. As default setting, the parameter values are randomly chosen during load execution. However, there are also parameter values which cannot be reused (e.g., identifiers generated during load test execution). A limitation of the JMeter Test Plan Generation process is that the values of these parameters cannot be generated automatically. Thus, these parameter values must be identified and specified manually by the load tester. For instance, when it is necessary to specify an item for a delete request on the protocol level. These identifiers can either be generated randomly or extracted using regular expressions or XPath extractors. In case Web site are tested, these IDs can be extracted from the HTML code.

The Workload Intensity is stored as a formula string in the *Session Arrival Controller* sub-component of a Test Plan’s (unique) Markov Session Controller. That controller additionally includes a table for Behavior Mix frequencies, to be filled with appropriate values of the input WESSBAS-DSL instance. Behavior Models are stored separately in CSV files, which are referred by the frequency table of the Markov Session Controller. In addition to the Test Plan elements that result from the core transformation process for a given WESSBAS-DSL instance, several JMeter elements are added to a generated Test Plan by default. This step is required for making a Test Plan’s structure accessible for the JMeter tool and providing additional functionality, such as handling of HTTP session cookies. Figure 9 shows the mapping of WESSBAS-DSL instances to JMeter Test Plan elements. Currently, the Test Plan structure is predefined, targeting HTTP-based tests only; an appropriate mechanism for specifying alternative structures, particularly for different types of requests, denotes a future work issue.

## Transformation to performance models

This section explains the proof-of-concept transformation of WESSBAS-DSL instances to workload specifications of the Palladio Component Model (PCM). First, Sect. 6.1 gives a short overview of PCM, followed by the description of how the system-specific parts of performance model are generated (see Sect. 6.2). Finally, Sect. 6.3 depicts how WESSBAS-DSL instances are transformed to workload specifications of PCM.

### Palladio Component Model

PCM is a domain-specific modeling language that allows the prediction of quality-of-service attributes (QoS) like response times, utilization, and throughput [8]. It is composed of five complementary model types. The central model type is the *Repository Model*, which models the software components, component operations, and the relations between them. The components can provide an interface to offer operations to other components or require interfaces to access operations from other components. The modeled components are assembled in a *System Model* to represent the application system. Resource containers (e.g., servers) and their associated hardware resources are modeled in the *Resource Environment Model*, whereas the *Allocation Model* defines the allocation of assembled components to the resource container. The *Usage Model* defines the workload of the system.

### Generation of performance models 

As our proposed approach focuses on the generation of PCM workload specifications, the system-specific parts of the model need to be created in a separate step. Since manual modeling requires too much effort, approaches that automatically extract PCM instance from design specification or running applications (e.g., [11, 15]) can be used to generate the system-specific part of the SUT.

We use the approach proposed by Brunnert et al. [15] to generate the system-specific parts of the performance model. A Java EE Servlet filter is used to collect data about the requests to Web components (i.e., Servlets, JavaServer Pages (JSP)). The data include the component invocations, the relations between them, and CPU resource demands for each request. Afterward, the performance model is created and the mean CPU demand per component invocation is integrated. We create the performance model on the level of requests to the web components only and do not split further into other components like Enterprise JavaBeans (EJBs). Thus, the resulting model is very simple. For each simulated request the average measured CPU time will be used for the performance prediction. Further details on the performance model generation approach are presented in [15].

### Transformation

The PCM Usage Model offers only basic support for modeling complex workloads: that is, the Usage Model does not allow the modeling of arbitrary usage flows. Each element can only have one incoming and one outgoing edge. Branches can be modeled with branch actions composed of multiple branch transitions, but it is not possible to interconnect elements of different branch transitions with each other. Thus, control flows like nested loops or connections between elements of one branch transition with elements of other branch transition cannot be modeled. Furthermore, the Usage Models grow in complexity for larger workloads, due to the lack of concepts enabling reuse. Consequently, we cannot transform the WESSBAS-DSL solely to the Usage Model. We generate parts of the workload specification into the Repository Model (cf. [53]) as it does offer this kind of structuring. This violates the clear separation of the PCM models but reduces the complexity of the transformation considerably. Furthermore, using this approach we do not need to extend the PCM meta-model.

**Table 3:** Mapping of WESSBAS-DSL concepts to PCM Model elements

WESSBAS-DSL	PCM Model elements
Session Layer FSMs	Repository Model (Basic Component, RDSEFF)
Protocol Layer FSMs	Not required
Workload Intensity	Usage Model (Open / Closed Workload)
Behavior Models	Repository Model (Basic Component, RDSEFF)
Behavior Mix	Usage Model (Branch)
Input parameters	Not required
Guards and actions	Parameter

*RDSEFF* resource demanding service effect specification

**Fig. 10:**
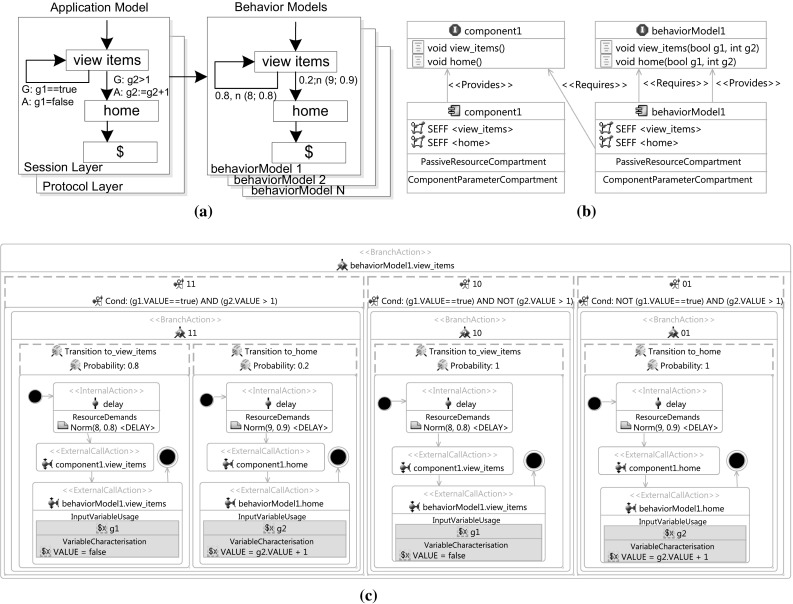
Exemplary transformation to PCM. **a** Workload Model example. **b** PCM Repository Model example. **c** RDSEFF of behaviorModel1.view_items

During the transformation, elements of the WESSBAS-DSL are mapped to elements of PCM as summarized in Table 3. The Protocol Layer and the input parameter cannot be modeled in PCM; this information is only required for load generators.

The GaAs and the probabilities cannot be modeled independently from each other in PCM. Therefore, the Behavior Models and the Session Layer are combined by modeling the GaAs of the Session Layer transitions to the transitions of the Behavior Model.

First, the PCM Repository Model generated by the performance model generator in the previous step is loaded and for each Behavior Model of the WESSBAS-DSL a new component with a corresponding interface used to represent the relationships between the components is generated. Furthermore, for each Markov State of a Behavior Model, a component operation as RDSEFF [8] is created. An RDSEFF describes the performance relevant behavior of component operation in a way similar to UML activity diagrams. The values of the guard conditions are passed using input parameter.

An example can be found in Fig. 10. The Workload Model in Fig. 10a will be first transformed to the PCM repository model (see Fig. 10b). *Component1* with the operations *view_items* and *home* is a component of the SUT already generated by the performance model generator. For *behaviorModel1*, a new component and an interface are created with the RDSEFFs *view_items* and *home*. The Behavior Model component requires the interface of *component1* as this component provides the system operations that are called during the transitions. Furthermore, *behaviorModel1* requires its own interface, as operations from the Behavior Models call themselves.

The transitions of the current Markov State to its successors are represented within each RDSEFF. In this way, the allowed sequence of service invocations is controlled by the Markov States themselves. Each RDSEFF consists of one branch. Within this branch, guards (if existing) represented as input parameters are first evaluated using guarded branch transitions. Afterward, for each guarded branch transition, a branch with probabilistic branch transitions to the next Markov States is modeled. In case a guarded branch transition is false, the probability of the remaining transitions must be adjusted to 100 %. As PCM cannot recalculate the probabilities based on the results of the conditions dynamically, all possible guard combination outcomes are calculated first for each RDSEFF. We exclude the case where all conditions are false, as in this case, no transition can be chosen and the execution terminates. Since no overlapping branching conditions are allowed in PCM, we ensure that only one of the conditions is true.

An RDSEFF example can be found in Fig. 10. As depicted in this figure, the RDSEFF for the Markov State *view_items* of the generated Behavior Model component *behaviorModel1* has three guarded branch transitions representing the possible guard combination. Assuming both guard conditions are true, the branch transition *to_view_items* has a probability of 80 % and the branch transition *to_home* has a probability of 20 %. In cases where guard $g2 > 1$ is false, the transition *to_home* cannot be executed. As a result, the probability of the transition *to_view_items* is increased to 100 % (respectively for transition *to_view_items*).

Each resulting branch transition specifies the call probability and contains three different actions:

1. The think time of this transition is modeled as specified in the WESSBAS-DSL using an *InternalAction* as a normal distribution with mean and deviation. In our example, the think time is specified as a normal distribution with mean and deviation. The normal think time can be less than zero as we use normal distributed think times. Therefore, zero is used when the value is negative.
2. The matching operation of the modeled system components is called as an *ExternalCallAction*. To identify the corresponding system operation, we use name mapping between the name of the system operation and the name of the Markov State. Only the operations of components that provide external system calls will be matched with the Markov State names. In the transition *to_view_items* of our example, the operation *view_items* of *component1* is called as it has the same name as the next Markov State *view_items*.
3. The RDSEFF of this Behavior Model component representing the next Markov State is called as *ExternalCallAction*. The values of the guard conditions are adapted according to the corresponding action; in the *to_view_items* transition of our example, the *view_items* state is called again and the value of *g1* is set to true. In the *to_home* transition, the state *home* is called and the value of *g2* is increased by one. In this way, the RDSEFFs of the Behavior Model component call themselves until a RDSEFF without successor is reached. Then, no further call is modeled, and the sequence terminates. In our example, *home* does not have a successor as there is only a transition to the exit state.

After creating the Behavior Model components in the Repository Model, each newly created component is allocated to the System Model and correspondingly to the Allocation Model. A new Usage Model is generated with one probabilistic branch representing the Behavior Mix. For each created Behavior Model component, a branch transition with the relative frequency as call probability is created. The initial Markov State of the Behavior Model is called within this transition. Finally, the Workload Intensity is modeled as closed workload with (i) the population representing the number of active sessions and (ii) the think time between the end and the start of a new session.

## Evaluation

During evaluation, we apply our proposed extraction approach and tooling to the industry-standard benchmark SPECjEnterprise2010^5^ and to the World Cup 1998 access logs [33]. This serves as an investigation of (i) the representativeness of the extracted workload specifications (quantitative) and (ii) the practicality of the approach and tooling support (qualitative).

### Research questions and methodology

We particularly investigate the following five research questions in order to evaluate our proposed approach:

- *RQ 1: How accurately do the clustering results match the input Behavior Mix?* The accuracy of the clustering is evaluated based on the fraction of misclassified sessions over all classifications of the clustering for benchmark runs using different Behavior Mix settings (see Sect. 7.4.1). To answer this question, classified sessions are required.
- *RQ 2: What is the impact of the clustering results on the workload characteristics of the executed and predicted workload?* First, JMeter and PCM instances are extracted. The JMeter test plans are executed on the SUT and the PCM instances are simulated. Afterward, the impact of the clustering on the workload characteristics is evaluated based on: (i) three session-based metrics, namely session length distribution (as number of requests per sessions), session duration, and number of distinct session types; (ii) a request-based metric, namely the relative invocation frequency of all request types. Conclusions about the arrival rates of requests can be drawn by looking at the invocation frequencies of requests (see Sects. 7.4.2, 7.4.3).
- *RQ 3: How accurately do the performance characteristics of the production system/SUT match the performance characteristics using the generated and predicted workload?* The accuracy of the performance characteristics using the generated and predicted workload is evaluated based on CPU utilization, response times, and heap usage. The heap usage is only evaluated for the measured and extracted workload, as it cannot be modeled and predicted using PCM (see Sect. 7.4.4).
- *RQ 4: How accurately do the workload and performance characteristics match when applying different workload settings to the extracted workload?* In test environments, it is often of interest to evaluate the impact of different workload settings like increasing Workload Intensity or different Behavior Mixes. Therefore, we evaluate workload and performance characteristics when these two workload settings are changed. We use the extracted workload from RQ 2 and RQ 3 and change the Workload Intensity and mix. The results will then be again compared with measured characteristics extracted from the original workload (see Sect. 7.4.5).
- *RQ 5: What is the impact of GaAs on the workload and performance characteristics? * The impact of the GaAs will be evaluated by executing workloads with and without the use of GaAs (see Sect. 7.4.6).

Ideally, these questions should be answered using logs of a real-world system to obtain production workloads with corresponding performance measurements and a test environment for load testing. Some non-synthetic logs of real-world system are publicly available and have been used by researchers. However, we do not have performance measurements of these systems as well. Furthermore, we have no access to test environments of these systems to evaluate the extracted JMeter Test Plans. Thus, we can use these publicly available logs only to evaluate RQ2. Using synthetic logs imposes a threat to external validity, and performance measurements would also be not available. As a result, laboratory experiments under controlled conditions are the best option for us, as we are able to evaluate all RQs. Therefore, we select an industry-standard benchmark that includes a representative workload profile.

To evaluate the approach with non-synthetic logs, we use the World Cup 1998 access logs. As the sessions of these logs are not classified and as we do not have access to the Web application to analyze performance characteristics, we can use these logs only for the evaluation of RQ 2. We extract a WESSBAS-DSL instance from the logs and transform it to a JMeter Test Plan and to a PCM instance. As the World Cup Web site is no longer available, we developed and instrumented a mock-up Website that has no functionality and accepts all kinds of requests. We execute the extracted JMeter Test Plan on this Web site, which enables us to measure the workload characteristics of the extracted JMeter Test Plan. The system-specific part of the PCM Model is modeled manually and consists of one system component and default resource demands (see Fig. 10).

An instrumented version of SPECjEnterprise2010 is used for the evaluation of all five RQs. Using this application, we are able to measure workload and performance characteristics. We executed the application with four different Behavior Mixes to obtain session logs. Based on these logs, the clustering is executed and evaluated. For the Behavior Mix extraction, we applied different configurations of the X-means clustering. Afterward, a WESSBAS-DSL instance is automatically generated from the obtained Behavior Models as described in Sect. 4.6. The resulting GaAs are shown in the Appendix (Table 10). The transformation from the instances to JMeter Test Plans is performed according to Sect. 5. The transformation to workload specifications of PCM is applied as described in Sect. 6. The extracted workload is executed on the same SPECjEnterprise2010 deployment in order to evaluate the workload and performance characteristics. Hence, the same session log analysis infrastructure as used for measuring the workload can be applied.

### Fifa World Cup 1998 access logs

In order to evaluate RQ 2 with non-synthetic access logs, we used the World Cup 98 Web site logs [2, 33]. The logs were recorded by a typical web server over a period of about three months. Each log entry contains a unique client identifier which may also be a proxy. To identify client sessions, we used a common timeout value of 30 minutes [22] as threshold between two user requests (see Sect. 4.1). During a session, the clients move from one page to another following navigation links. The URLs of the Web site follow the form “/language/category/subcategory/page_name” where category and subcategory are not always used. For instance “/english/competition/statistics.htm”, “/english/index. html”, and “/english/history/past_cups/uruguay30. html” are typical URLs.

The application consists of over 32,000 pages [22]. We grouped the links into the categories and subcategories resulting in 25 groups each of which corresponds to a service. When no category and subcategory is used (e.g., “/english/index.html”), we use the term “mainLevel” as category.

### SPECjEnterprise2010 deployment

The SPECjEnterprise2010 industry benchmark deployment is used for the evaluation of the proposed approach. SPECjEnterprise2010 is a Java EE application representing a business case combining customer relationship management (CRM), supply chain management (SCM), and manufacturing. It includes a workload specification and a dataset which is needed for the execution of load tests. The workload is generated by the Faban Harness and Benchmark Driver.^6^ The benchmark consists of three different application domains; namely *Orders domain* (CRM), *Manufacturing domain*, and *Supplier domain* (SCM). The *Orders domain* (CRM) provides a Web-based user interface representing a standard e-commerce application with product information and a shopping cart. It drives the demand to the *Manufacturing domain*, which simulates production across different manufacturing plants. The task of the *Supplier domain* (SCM) is to order new parts for the Manufacturing domain. In this work, we consider only the Orders domain, which represents a typical Web-based application providing e-commerce functionality to customers; in this case automobile dealers. Using this application, customers are able to purchase and sell cars, to manage their accounts and dealership inventory, and to browse the catalog of cars. The Orders domain runs independently from the other two domains, as they are mainly intended to be used as (Web-)service by other applications. It represents the production system/SUT.

**Fig. 11:**
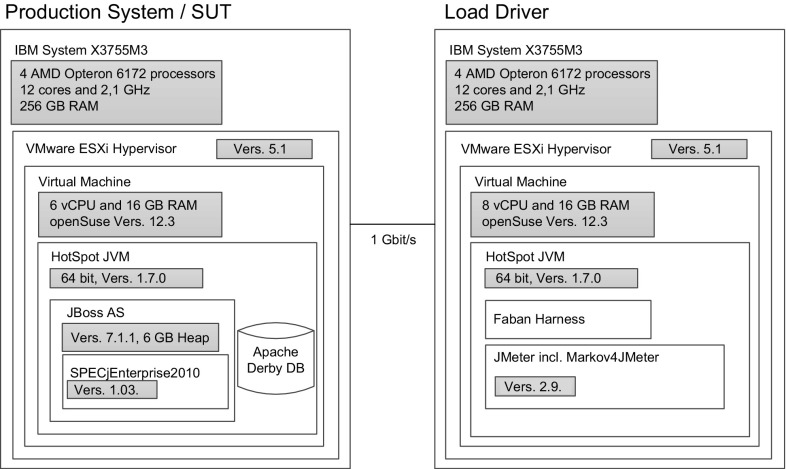
Hardware and software infrastructure

#### Hardware infrastructure

The SUT and the Dealer Driver are deployed on separate virtual machines (VM), linked by a 1 GBit/s network (see Fig. 11). The SUT is deployed on an IBM System X3755M3 server with 6 virtual CPUs and 16 GB RAM. The Dealer Drivers also run on an IBM System X3755M3 server VM with 8 virtual CPUs and 16 GB RAM. For the JMeter load test, we used 3 VMs (800 User) and 4 VMs (1200 User). The application server is JBoss 7.1.1. using the Java EE 6 full profile with 6 GB heap allocated. As persistence layer, an Apache Derby DB is used running in the same JVM as the JBoss application server. Both systems use openSUSE operating system in version 12.3 and are executed on a 64-bit OpenJDK 1.7.0 Server Java VM in version 1.7.0.

**Fig. 12:**
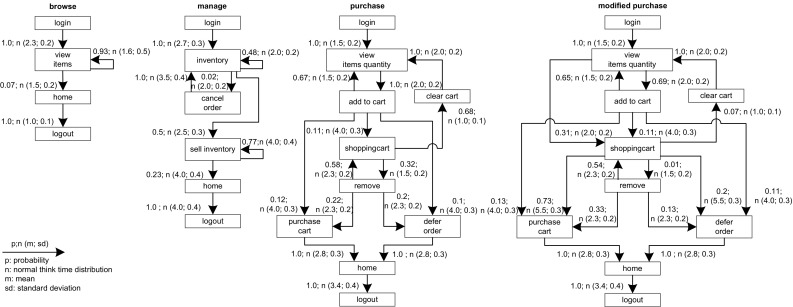
SPECjEnterprise2010 transactions *Browse*, *Manage*, and *Purchase* as Behavior Models. The transaction *Modified Purchase* is used for the evaluation of RQ 5 (see Sect. 7.4.6). The probabilities are rounded to two decimal places and the mean and standard deviation of the think times to one decimal place

#### Workload description

SPECjEnterprise2010 defines three different transaction types which are executed by automobile dealers: *Browse (B)*, *Manage (M)*, and *Purchase (P)*. Within the transaction type Browse, the benchmark driver navigates to the catalog of available cars and browses the catalog for a constant number of thirteen times. Manage describes a scenario during which open orders are canceled and vehicles are sold. In the more complex transaction type Purchase, orders are placed and immediately purchased or deferred. Either the shopping cart is cleared or items are removed one by one until only one item remains. Each of these transaction types corresponds to a sequence of HTTP requests. The workload in the Faban dealer driver is not defined in a probabilistic way and only a few of the HTTP requests are generated in a probabilistic way.

SPECjEnterprise2010 defines a total of 13 different HTTP request types, using a request parameter called *action*. We additionally split the request type called *View_Items* into two different request types as it executes two different use cases resulting in different resource demands; one request type is *View_Items* and the other is *View_Items_Quantity*. In the first use case, *View_Items* is called to browse the catalog of available cars. In the second use case, only one specific item of the catalog is selected.

Within the original dealer driver, no think times are defined between the execution of the HTTP actions, i.e., each HTTP action is executed directly after its previous request has been completed. Therefore, we added think times between these actions as Gaussian distribution with mean and standard deviation. The think times are randomly specified between mean values of 1–4 s. Figure 12 depicts the structure of the three transaction types as Behavior Models, obtained by applying our WESSBAS extraction approach including the transition probabilities and the specified think times.

In the original benchmark workload, automobile dealers log in to the system, execute *multiple instances* of the three transactions types, and log out. Each of the three transaction types is executed with a specified probability. The standard transaction mix is 50 % Browse, 25 % Manage, and 25 % Purchase. We modified the dealer driver such that *each* transaction starts with a login and ends with a logout. In this way, each transaction corresponds to a unique session and the transaction mix corresponds to the Behavior Mix. As a result, the transaction types define the different navigational patterns.

#### Benchmark execution and monitoring

Four different transaction mixes are used to evaluate the proposed approach. For each mix, one of the transaction types is executed with a probability of 50 % and the other two with 25 % each. Additionally, a mix is chosen where the proportions of the transaction types are equal. A load of 800 concurrent users is executed, resulting in a moderate CPU utilization of the SUT of approximately 30 %. Each benchmark run is executed for twelve minutes after a three minute ramp-up phase and before a three minute ramp-down phase. We extract the system-specific parts of the performance model (as described in Sect. 6.2) using the original workload mix once. This part of the performance model will be reused during the evaluation.

The SUT is instrumented using Kieker [52] to obtain the raw session information. Each request is recorded and afterward transformed to a session log (see Sect. 4.1). During the transformation, we only take complete sessions during steady state into account; meaning, sessions starting with a login request after the ramp-up phase and ending with a logout request before the ramp-down phase. Thus, incomplete sessions are removed. In order to be able to evaluate the clustering results of the transaction types, the name of the transaction type is added as an additional parameter to the login HTTP action.

We use the same procedure to predict the performance with PCM. However, as PCM does not provide a unique session identifier for interrelated requests, we cannot remove incomplete sessions during steady state. As a result, the predicted request counts are a little bit higher than the measured ones.

**Table 4:** Clustering results

TM	T	X-means (min 3 cluster, max 3 cluster)								X-means (min 2 cluster, max 20 cluster)								N
		ED				NED				ED			NED					
		C1	C2	C3	MC	C1	C2	C3	MC	C1	C2	MC	C1	C2	C3	C4	MC	
50	B	10,346	0	0	3.03 %	10,346	0	0	0 %	10,346	0	24.64 %	0	10,346	0	0	0.96 %	19,890
25	M	0	0	5230		0	0	5230		0	5230		0	0	199	5031		
25	P	0	4468	625		0	5093	0		0	5093		5093	0	0	0		
25	B	0	0	5077	16.48 %	5077	0	0	0 %	0	5077	24.91 %	0	0	0	5077	0.89 %	20,395
25	M	0	5081	0		0	0	5081		5081	0		182	4899	0	0		
50	P	6875	3362	0		0	10,237	0		10,237	0		0	0	10,237	0		
25	B	0	5092	0	2.99 %	5092	0	0	0 %	5092	0	24.72 %	0	0	0	5092	1.62 %	20,125
50	M	10,058	0	0		0	0	10,058		0	10,058		326	9732	0	0		
25	P	586	0	4389		0	4975	0		0	4975		0	0	4975	0		
34	B	0	0	6917	4.1 %	0	0	6917	0 %	0	6917	32.9 %	0	0	0	6917	1.16 %	20,470
33	M	6818	0	0		6818	0	0		6818	0		6581	237	0	0		
33	P	840	5895	0		0	6735	0		6735	0		0	0	6735	0		

### Evaluation results

#### Accuracy of clustering

The evaluation of clustering accuracy (RQ 1) is split into two steps. In the first step, the accuracy of the clustering is determined based on the assumption that the number of resulting clusters is known in advance. For this reason, the number of resulting clusters is fixed to three. As the number of clusters is usually not known in advance, we let the X-means algorithm determine that number in a second step. Since the seed value for the random selection of the initial centroids can have a high impact on the clustering results, multiple clustering runs are executed with different seed values between one and twelve. Afterward, the run with the lowest sum of squared error value [42] is selected.

The results of the clustering are presented in Table 4. For each transaction mix (TM), the clustering shows for each transaction type (T) the cluster (C$_{x}$) to which a transaction is assigned, and the percentage of misclassified (MC) transactions. The left side of the table shows the results of exactly three predefined clusters (step one); the right side shows the results of letting X-means determine the number of clusters between two and twenty (step 2). The number of transactions (N) clustered for each transaction mix is around 20, 000.

The results of using exactly three clusters indicate that the clustering with use of normalized Euclidean distance (NED) is able to cluster all transactions correctly (100 %), resulting in the Behavior Models shown in Fig. 12. The clustering using Euclidean distance (ED) without normalization classifies the transactions Browse and Manage correctly, whereas a fraction of transactions of type Purchase is assigned mistakenly to the same cluster as the Manage transactions. In the second transaction mix, the fraction of Purchase transactions is higher than in the other mixes. Hence, the percentage of misclassified transactions is high (16.48 %). As a result, the clustering using ED is not able to cluster all transactions correctly, although each transaction comprises unique states.

The clustering without predefining the correct number of clusters always results in two clusters using ED and four clusters using NED. As clustering with ED always merges transactions of type Purchase and Manage, the percentage of misclassified transactions is between 25 and 33 %. It is assumed that the transaction type with the lower number of instances merged within one cluster counts as misclassified. The clustering using NED always correctly classifies Browse and Purchase transactions, whereas Manage transactions are always split into two clusters. Hence, the percentage of misclassified transactions is very low (around 1 %) for all transaction mixes.

Transactions of type Browse seem to be homogeneous in a way that they are clustered correctly among all clustering runs. This can be explained as Browse transactions are executed with a constant number of actions without probabilistic behavior. NED is better suited for clustering the different transaction types than the non-normalized version. The normalization has the effect that high transaction counts, and therefore also the session lengths have a lower impact on the clustering. Thus, the structure of the transactions in terms of the number of different HTTP requests grows in significance. As each of the three transaction types consists of different HTTP request types (except for *login*, *home* and *logout*), the clustering results are significantly better.

#### Accuracy of World Cup 1998 workload characteristics

We investigate research question RQ 2, by analyzing the impact of the clustering results on server-side session-based and request-based metrics (mentioned in Sect. 7.1) for the original measurements with the corresponding metrics obtained by executing extracted workload specifications using JMeter and PCM. In this section we present the results of the non-synthetic World Cup 1998 logs. In Sect. 7.4.3 the accuracy of the workload characteristics of SPECjEnterprise2010 is presented.

We analyzed the logs of day 42 of the World Cup in English language. We identified 53,644 sessions and in total 511,824 page requests. The logs are analyzed as described in Sect. 7.2, and a WESSBAS instance is created. During the transformation no GaAs could be identified, as the Website is created in way that each Web site can be accessed by all other Web sites. We clustered the logs using X-means clustering with a minimum of 2 clusters and a maximum of 20 clusters with NED distance metric resulting in four clusters. The relative frequency of each request type per cluster can be found in Table 5.

The average session lengths within the cluster range from 7.58 (C4) to 13.28 (C2) requests, and the number of sessions per cluster ranges from 3,447 (C3) to 21,205 (C2). The clusters differentiate primarily in the four request groups *mainlevel*, */teams*, */news*, and */competition*, which make up 86 % of all requests.

The users of the first cluster search mainly for teams and navigate less on the main level. In the second cluster, the users navigate on the main level and on teams’ pages. In the third cluster, the users request news pages and fewer on pages from the request type teams. The fourth cluster contains mainly users browsing on the main level and on pages of category competition. Users of the fourth cluster have the lowest session length. Overall, we can see that the clustering is able to identify different kinds of user groups.

**Table 5:** World Cup 1998 Logs: relative frequency of each request category per cluster

Category	C1	C2	C3	C4
Mainlevel	11.08 %	31.79 %	35.22 %	55.67 %
/teams	60.96 %	48.10 %	14.56 %	11.68 %
/news	14.03 %	8.22 %	18.07 %	8.26 %
/competition	5.34 %	5.81 %	8.02 %	16.11 %
/enfetes	0.96 %	1.28 %	3.05 %	1.43 %
/playing	0.98 %	0.76 %	3.27 %	1.11 %
/playing/download	0.61 %	0.35 %	2.77 %	0.59 %
/history/past	1.00 %	0.52 %	2.46 %	0.62 %
/tickets	0.71 %	0.50 %	2.29 %	0.79 %
/playing/mascot	0.52 %	0.46 %	2.14 %	0.66 %
/venues	0.39 %	0.30 %	1.31 %	0.49 %
/venues/cities	0.82 %	0.32 %	1.15 %	0.36 %
/history	0.38 %	0.28 %	1.00 %	0.33 %
/help	0.24 %	0.23 %	0.74 %	0.37 %
/hosts/cfo	0.23 %	0.18 %	0.77 %	0.28 %
/member	0.16 %	0.21 %	0.63 %	0.34 %
/hosts/suppliers	0.49 %	0.07 %	0.66 %	0.34 %
/venues/venues	0.46 %	0.23 %	0.59 %	0.23 %
/history/history	0.19 %	0.14 %	0.51 %	0.16 %
/individuals	0.31 %	0.16 %	0.29 %	0.05 %
/hosts/fifa	0.02 %	0.05 %	0.16 %	0.05 %
/hosts/sponsors	0.04 %	0.02 %	0.15 %	0.04 %
/history/reading	0.02 %	0.02 %	0.10 %	0.03 %
/hosts/fff	0.02 %	0.01 %	0.06 %	0.02 %
/playing/rules	0.01 %	0.00 %	0.01 %	0.00 %
# sessions	3447	8492	21,205	20,500
Avg session length	8.43	13.28	10.12	7.58

The WESSBAS instance is then transformed into a JMeter Test Plan and executed against the mock-up Web site (see Sect. 7.1) to measure the workload characteristics. Moreover, a PCM instance will be generated and simulated. The results are presented in the following.

**Table 6:** Summary statistics of session lengths

	Min	*Q* $_1$	Med.	Mean	CI$_{0.95}$	*Q* $_{3}$	Max	*N*
*Orig.*	1	4	6	9.54	[9.37, 9.72]	11	1972	53,644
*NED-4*	1	3	7	9.51	[9.43, 9.58]	13	91	53,326

*Session-based metrics* The session-based statistics are only compared against JMeter metrics as PCM does not generate unique identifiers for interrelated user actions. The evaluated session statistics can be found in Table 6.

The mean values and the 0.95 confidence interval indicate that both distributions are very similar. The number of distinct sessions of the extracted workload is with 27,548 also similar to 22,605 of the original workload. The difference in the number of distinct sessions comes from the fact that within the original workload, sessions with a very high number of user requests have been measured; at maximum 1972 in the original workload compared to 91 in the extracted workload. This can be explained as the unique client identifier in the original non-synthetic access logs could also include requests from a proxy comprising multiple users. In contrast, in the extracted workload the session lengths are evenly distributed.

Summarizing, the session-based metrics are similar and differentiate mainly by the fact that the original workload contains very long sessions caused by proxies.

*Request counts* In Table 7, the request counts of the measured (Orig.) are compared to the request counts of the executed (JMeter) and simulated (PCM) workloads. For the request counts, an almost exact match can be found for the 25 request groups. The deviation in the form of the sum of squared errors is zero. Thus, the server-side request counts are representative compared to the original workload.

**Table 7:** Absolute and relative (Rel.) request counts

Category	Orig.	JMeter	PCM	Rel.
Mainlevel	201,167	199,569	199,165	0.39
/teams	121,354	120,043	119,871	0.24
/news	64,966	64,151	64,006	0.13
/competition	50,354	50,134	49,135	0.10
/enfetes	10,503	10,249	10,256	0.02
/playing	9875	10,043	9866	0.02
/playing/download	7429	7449	7265	0.01
/history/past	7096	7066	6813	0.01
/tickets	6922	6708	6791	0.01
/playing/mascot	6299	6134	6495	0.01
/venues	4014	3928	3929	0.01
/venues/cities	3633	3661	3726	0.01
/history	3088	2983	3081	0.01
/help	2505	2381	2449	0.00
/hosts/cfo	2340	2333	2296	0.00
/member	2165	2174	2203	0.00
/hosts/suppliers	2164	2168	2061	0.00
/venues/venues	2008	2063	2009	0.00
/history/history	1557	1400	1633	0.00
/individuals	950	863	974	0.00
/hosts/fifa	489	509	471	0.00
/hosts/sponsors	428	458	394	0.00
/history/reading	288	264	301	0.00
/hosts/fff	189	222	153	0.00
/playing/rules	41	49	31	0.00
Sum	511,824	507,002	505,374	1.00

#### Accuracy of SPECjEnterprise2010 workload characteristics

In this section, we also evaluate RQ 2, but this time with the synthetic logs generated by SPECjEnterprise2010 which serves to increase the external validity. The session-based statistics are again compared only against JMeter metrics (see Sect. 7.4.2). We present the results of the original benchmark Behavior Mix (25 % P, 50 % B, and 25 % M), using the X-means clustering algorithms results with 2 (ED), 3 (NED), and 4 (NED) clusters (entries for the bottom TM in Table 4).

**Fig. 13:**
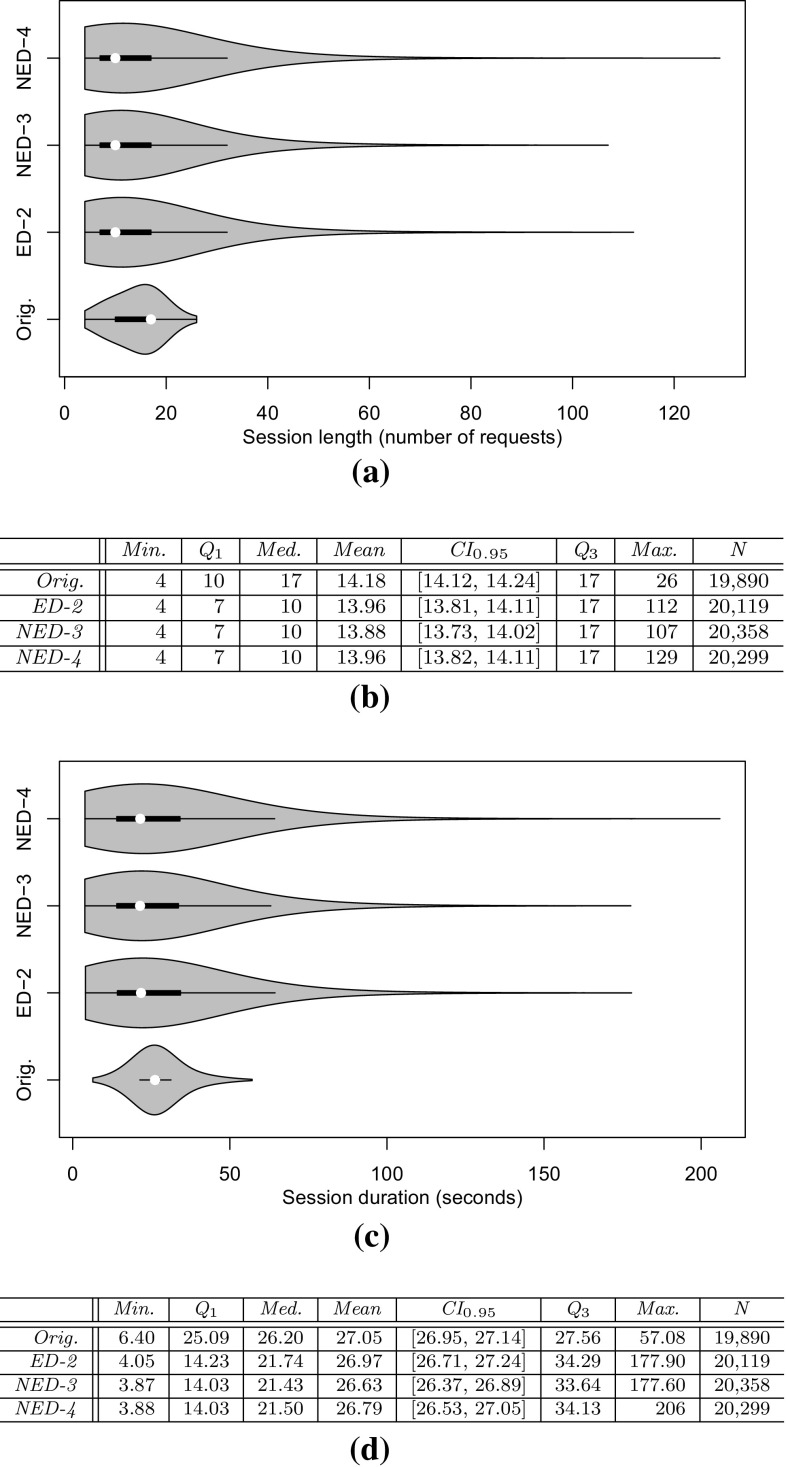
800U-50B/25P/25M: Session length and duration statistics for the original workload (Orig.) and the synthetic workloads (ED-2, NED-3, NED-4). **a** Violin plot of session lengths. **b** Summary statistics of session lengths. **c** Violin plot of session durations. **d** Summary statistics of session durations (in s)

*Session-based metrics* Statistics about the session length distributions of the original and the three synthetic workloads are shown in Fig. 13. Figure 13a depicts the four distributions as violin plots. Looking only at the mean values and the 0.95 confidence interval (Fig. 13b), one may conclude that the session length distributions of the three synthetic workloads exactly match the distribution of the original workload. However, particularly the violin plot (Fig. 13a) indicates that the synthetic distributions are similar but differ considerably from the original workload.

The quartile-based statistics in Fig. 13b confirm this observation. The same observation can be made for the distribution of session durations in seconds (Fig. 13c, d). Very long sessions for the synthetic workloads are generated. While for the original workload the longest sessions comprise 26 requests, the synthetic sessions reach maximums of 112, 107, and 129.

In the original workload, we identified 78 distinct sessions. The number of distinct sessions in the synthetic workloads is considerably higher, namely 1056 (2 clusters), 1004 (3 clusters), 960 (4 clusters). The relatively low number of distinct session types is caused by the fact that the original SPECjEnterprise2010 workload contains few probabilistic elements, which are all bounded in the number of maximum iterations (cf. Sect. 7.3.2). For instance, the *view_items* request in the browse transaction is executed exactly thirteen times. Hence, the maximum number of possible distinct sessions is countable. In our previous work [51] the number of distinct sessions is around 2000. The number of distinct sessions is lower in this paper as GaAs reduce the number of invalid sessions.

The described session length distributions of the synthetic workloads imply the high number of distinct sessions. Inspecting the structure of the synthetic sessions, we observed the following recurring patterns: (i) *sell inventory*+, (ii) *inventory*+, (iii) *view items*+, (iv) (*view items quantity*, *add to cart*)+, (v) (*view items quantity*, *add to cart*,* shopping cart*, *clear cart*)+. These patterns can be explained by the corresponding transitions with high probabilities already indicated by the probabilities of the original workload depicted in Fig. 12.

Considering the setting for SPECjEnterprise2010, the following conclusions can be drawn about the impact of the clustering results on the session-based metrics session length and number of distinct session types. Firstly, no statistically significant differences between the synthetic workloads for two, three, and four clusters in the summary statistics from Fig. 13 can be observed. Secondly, both the session length distributions and the number of distinct sessions deviate from the characteristics of the original workload. Thirdly, the deviation of the session length distributions is mainly caused by a number of synthetic long sessions. Lastly, the mean value shows no statistically significant difference.

*Request counts* Table 8 depicts statistics about the frequency of invoked requests using JMeter and PCM, based on the absolute numbers of requests to the 14 SPECjEnterprise2010 request types. We compared the request counts of the original workload with the request counts of the three different clustering settings executed with JMeter and simulated with PCM.

As in Sect. 7.4.2, an almost exact match of the relative frequencies could be observed. Hence, from the server-perspective, the synthetic SPECjEnterprise2010 is representative in terms of the distributions of requests.

**Table 8:** 800U-50B/25P/25M: Request count statistics

Request	Orig.	ED-2	NED-3	NED-4	Rel.
*(a) Absolute and relative (Rel.) counts (JMeter)*					
1. Add to cart	20,625	21,474	21,129	21,217	0.07
2. Cancel order	191	198	176	168	0.00
3. Clear cart	1932	2129	2011	1976	0.01
4. Defer order	2236	2228	2218	2312	0.01
5. Home	19,371	20,119	20,358	20,299	0.07
6. Inventory	10,034	10,273	10,136	10,064	0.04
7. Login	19,890	20,119	20,358	20,299	0.07
8. Logout	19,372	20,119	20,358	20,299	0.07
9. Purchase cart	2682	2780	2873	2795	0.01
10. Remove	923	660	675	723	0.00
11. Sell inventory	21,949	22,703	21,854	21,653	0.08
12. Shopping cart	2855	2789	2686	2699	0.01
13. View items	139,370	133,766	136,529	137,723	0.49
14. View items quantity	20,625	21,474	21,129	21,217	0.07
*(b) Absolute and relative (Rel.) counts (PCM)*					
1. Add to cart	20,625	22,416	22,466	21,936	0.07
2. Cancel order	191	217	165	208	0.00
3. Clear cart	1932	2094	2222	2062	0.01
4. Defer order	2236	2425	2379	2275	0.01
5. Home	19,371	21,131	21,190	20,990	0.07
6. Inventory	10,034	10,703	10,656	10,932	0.04
7. Login	19,890	21,128	21,190	20,997	0.07
8. Logout	19,372	21,128	21,190	20,997	0.07
9. Purchase cart	2682	2806	2919	2840	0.01
10. Remove	923	711	713	692	0.00
11. Sell inventory	21,949	23,867	23,552	23,807	0.08
12. Shopping cart	2855	2808	2939	2755	0.01
13. View items	139,370	146,637	146,903	148,698	0.49
14. View items quantity	20,625	22,425	22,472	21,930	0.07

#### Accuracy of performance metrics

In this section, performance characteristics of the SUT using the original workload are compared with resulting performance characteristics using the extracted and simulated workload (RQ 3). We analyze the resulting CPU utilization, server-side response times per request type and the heap usage (only Faban compared to JMeter). The results using the original benchmark Behavior Mix (50 % B, 25 % P, and 25 % M) with 3 (NED) clusters are presented.

**Fig. 14:**
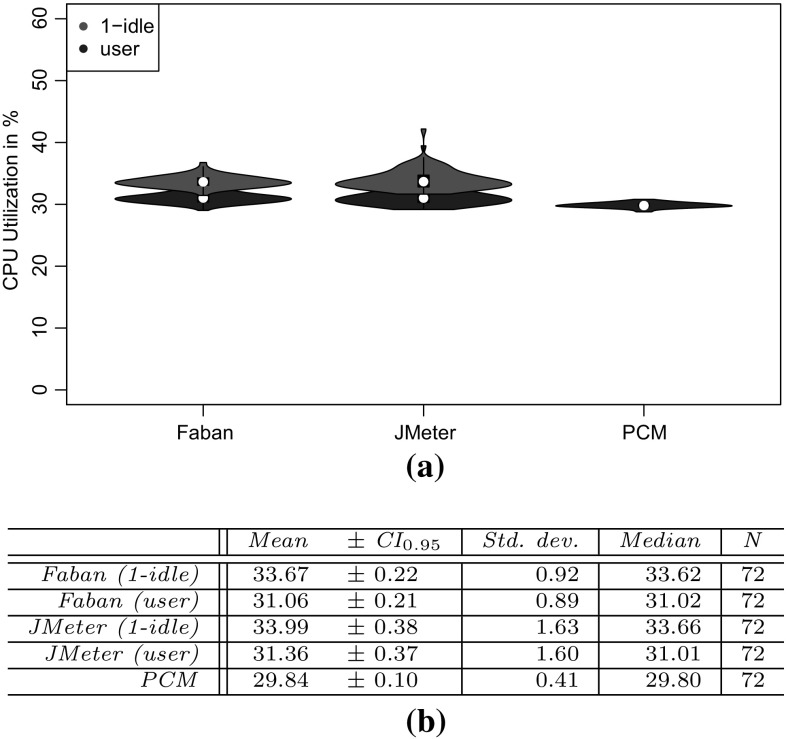
800U-50B/25P/25M: CPU utilization statistics. **a** Violin plots. **b** Summary statistics

*CPU utilization* Figure 14a illustrates the resulting CPU utilization using Faban Harness, JMeter, and PCM as violin plots. We measured the CPU utilization every 10 s using the Linux command line tool System Activity Reporter (SAR).^7^ The CPU utilization for the load driver is split into overall CPU utilization (1-idle) and user CPU utilization. As illustrated in Fig. 14b, the original workload using Faban resulted in a mean CPU utilization of 33.67 % (1-idle) and 31.06 % (user). The mean CPU utilization of JMeter is almost similar with 33.99 % (1-idle) and 31.36 % (user). However, the standard deviation using JMeter is higher. The predicted CPU utilization using PCM is 29.84 %. This is a prediction error of 11.4 % in relation to the overall utilization and of 3.92 % compared to the user CPU utilization. The difference can be explained by the fact that the used performance model generator [15] neglects the system utilization. The deviation of the predicted CPU demands is very low.

**Fig. 15:**
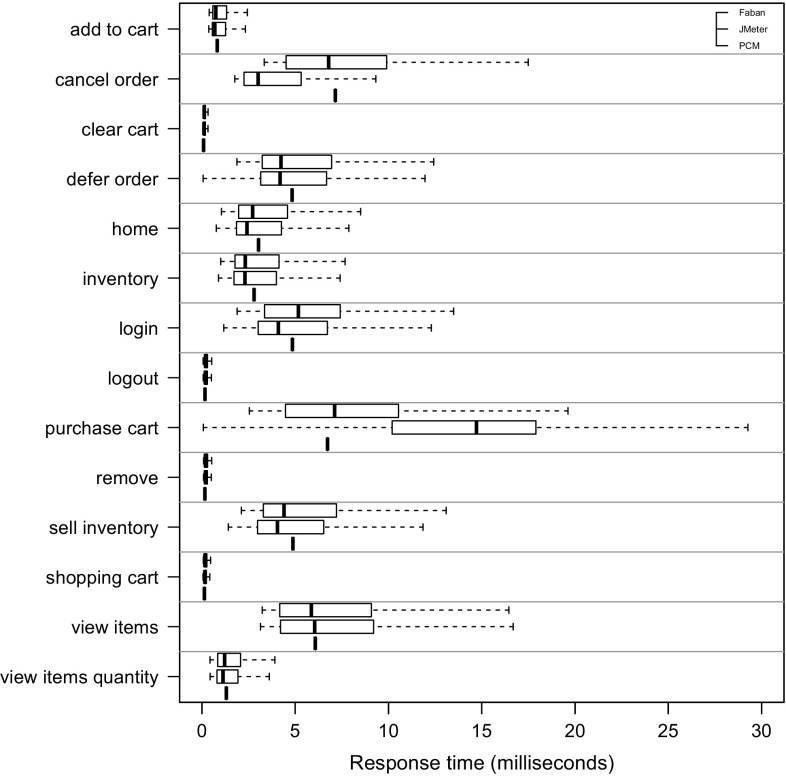
800U-50B/25P/25M: Server-side response time statistics

*Server-side response times* The resulting server-side response times in milliseconds per request type can be found in Fig. 15. The predicted mean response times using PCM are similar to the response times using Faban, but indicate very low response time deviations. As the generated performance model simulates average CPU demands per request type, the low deviation was expected (compare Sect. 6.2).

Comparing the response times of Faban with JMeter, the mean response times and the deviation are similar except for *purchase cart* and *cancel order*. The mean response times of *purchase cart* requests is with 16.85 ms considerably higher than the mean response times using the original workload (10.21 ms). Furthermore, the deviation is higher because the number of purchased items using JMeter can be considerably higher than in the original workload. In the original workload, a maximum of five items is purchased. As the extracted workloads are generated in a probabilistic way, the number of *add to cart* executions before the *purchase cart* request is not limited.

The reason why the mean response time of the *cancel order* requests is lower is similar. Before the request is executed, the original workload checks whether open orders exist. As JMeter generates these requests in a probabilistic way, it is possible that no open orders exist. Thus, the response times and the deviation are lower. This could be manually fixed by adding an additional guard condition into JMeter, which checks whether open orders exist. However, this kind of conditions cannot be extracted in an automatic way.

**Fig. 16:**
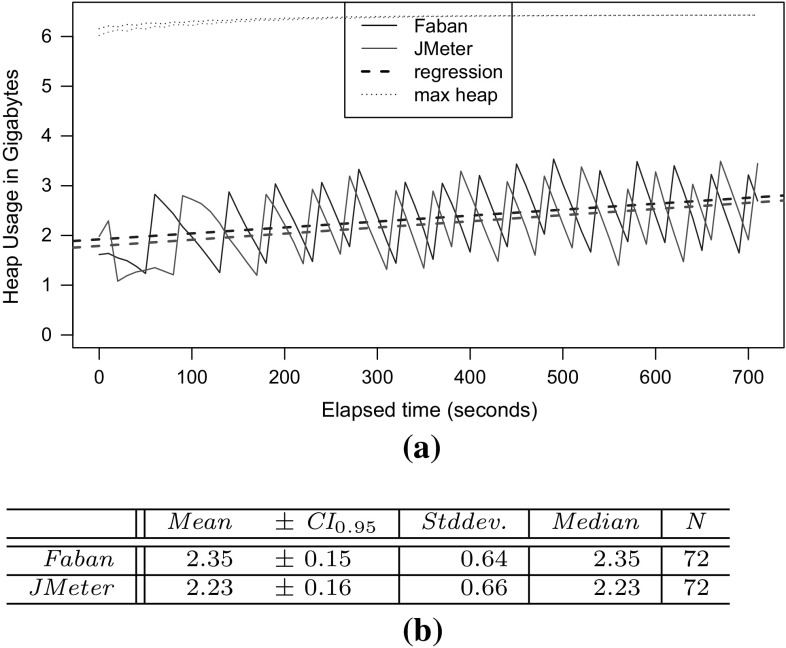
800U-50B/25P/25M: Memory usage statistics (with Faban initialization). **a** Usage over time. **b** Summary statistics

*Heap usage* We additionally analyzed the heap usage of the original workload compared to the extracted workload (see Fig. 16). As the Faban benchmark driver executes several read and write operations on the database (Faban initialization) before the ramp-up phase, the heap usage increases by approximately one gigabyte. In order to make the heap usage comparable, we also executed the Faban initialization phase before we started JMeter. As shown in Fig. 16b the resulting mean heap usage of Faban (2.35 GByte) and JMeter (2.23 GByte) are very similar. Additionally, the regression lines run in parallel (see Fig. 16a) on the same level.

#### Accuracy of changing workload settings

In this section we describe scenarios in which the settings of the extracted workloads are changed (RQ 4). These workloads are executed (respectively simulated) and then again compared with the workload and performance characteristics from a Faban run. In this way, we evaluate the accuracy of the extracted workload models under changed settings. In the first scenario we increase the Workload Intensity only, and in the second we increase the intensity and change the workload mix. In the following we present the relevant analysis.

*Increasing Workload Intensity* For the first scenario, we conducted the same experiment as before (standard benchmark mix, 3 (NED) clustering) but increased the Workload Intensity from 800 users to 1200 users. We first analyzed the workload characteristics. As the session-based and request-based metrics are almost identical to the run with 800 users (except for the higher number of sessions and requests) we will not present these metrics here.

**Fig. 17:**
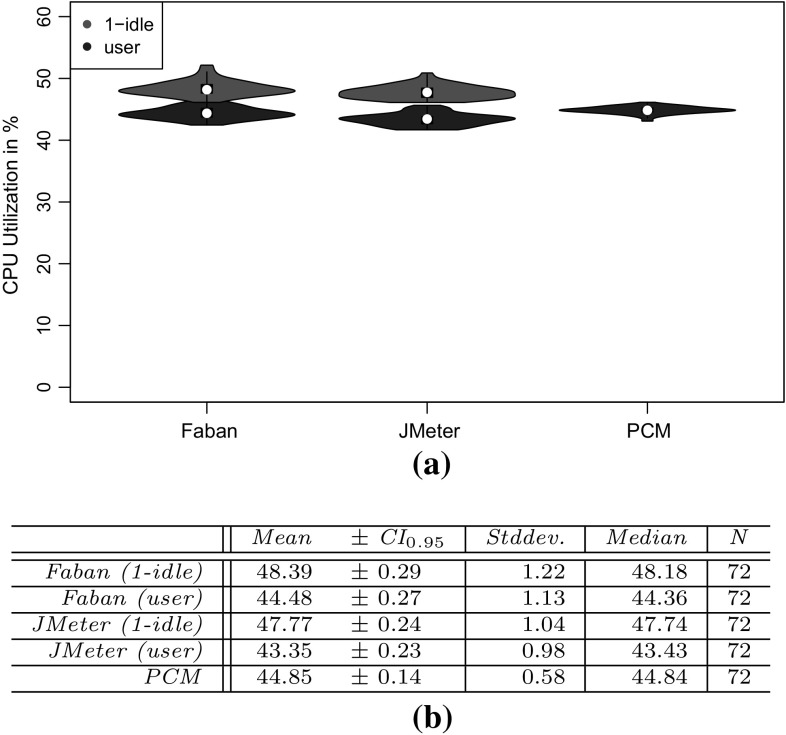
1200U_50B_25P_25M: CPU utilization statistics. **a** Violin plots. **b** Summary statistics

The CPU utilizations increased by approximately 15 % compared to the run with 800 users (Fig. 17). The statistics show that the mean CPU utilizations of Faban (48.39 %) and JMeter (47.77 %) are again quite similar. The relative prediction error of PCM compared to the overall utilization of Faban is 7.3 and 0.01 % for the user CPU utilization. Thus, the prediction error decreases compared to the run with 800 users.

**Fig. 18:**
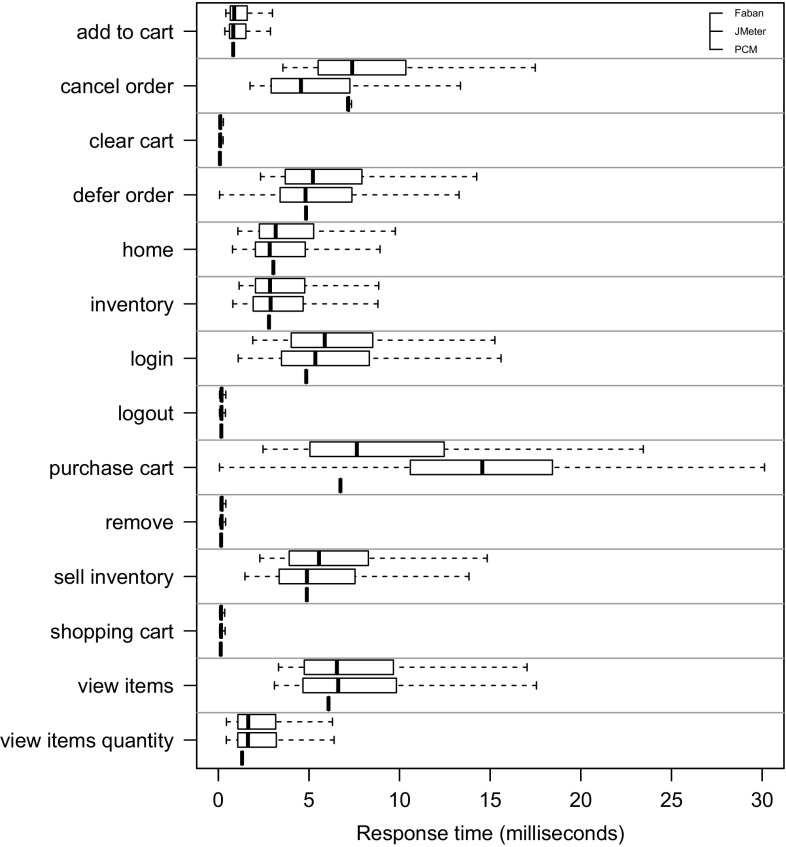
1200U-50B/25P/25M: Server-side response time statistics

The resulting server-side response times of Faban and JMeter increase by approximately 30 % (Fig. 18). Again, the response times are similar except for *cancel order* and *purchase cart*. The relative error for *purchase cart* requests is increased to 94 % compared to Faban. The predicted response times are only increased by approximately 2 % and are somewhat lower than the response times caused by the Faban load driver.

**Fig. 19:**
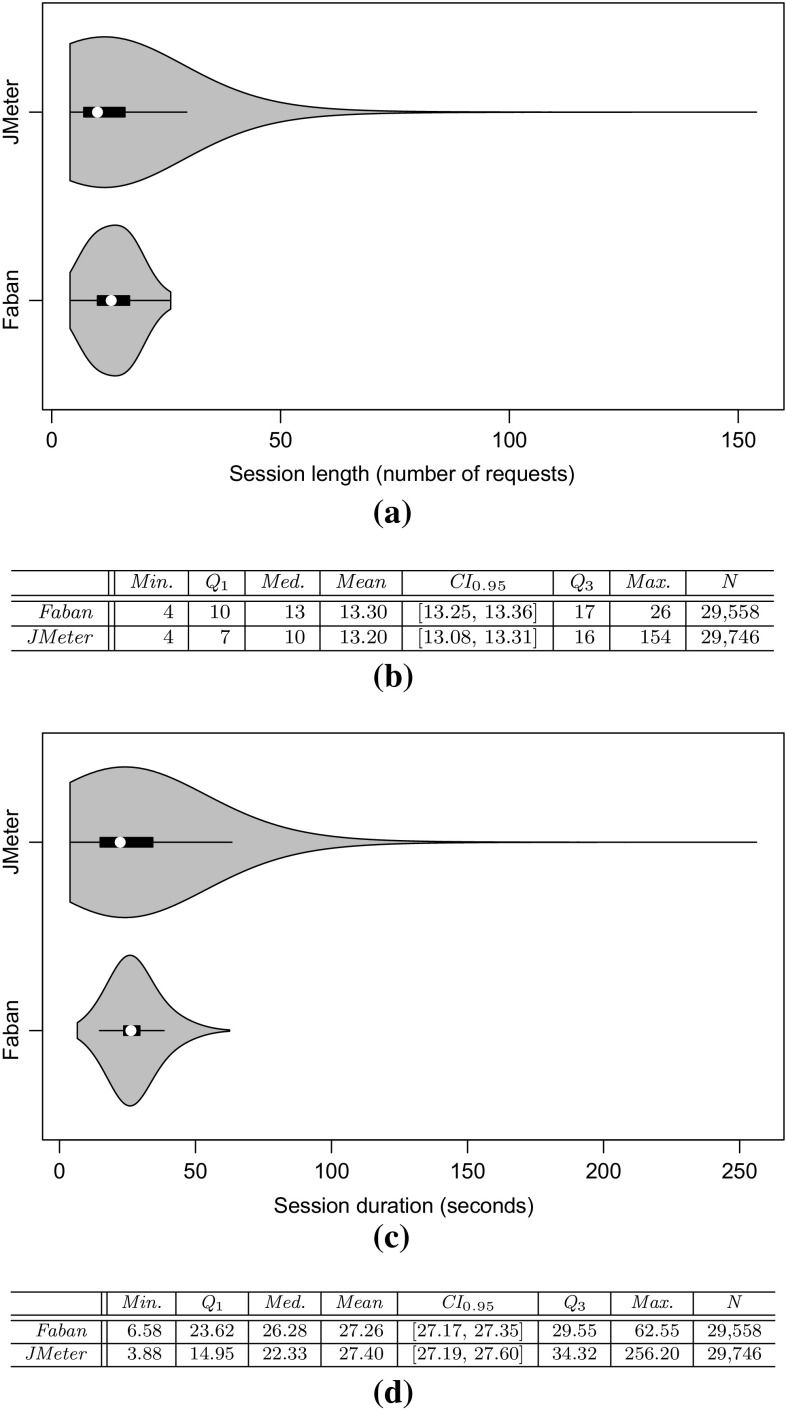
1200U-34B/33P/33M: Session length statistics. **a** Violin plot. **b** Summary statistics. **c** Violin plot. **d** Summary statistics

*Increasing Workload Intensity and changing Behavior Mix* In the second scenario, we additionally changed the Behavior Mix in a way that the proportion of the transaction types are of almost equal size (34 % B, 33 % P, and 33 % M).

**Fig. 20:**
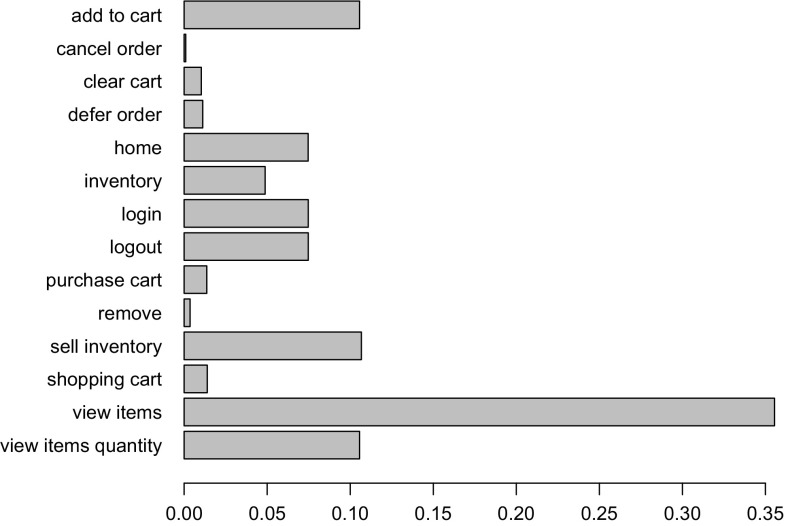
1200U-34B/33P/33M: Request count statistics. Relative counts (common to JMeter, Faban and PCM)

Figure 19 shows that the mean session length decreases slightly to a mean of 13.30 compared to the original workload mix (14.18). In contrast, the mean session duration increases slightly from 27.05 to 27.26 s. Again, metrics generated by Faban and JMeter are very similar. By comparing the request count statistics (Fig. 20) it can be seen that the relative error compared to the overall number of requests is again zero. This can be seen as the same bars are used for Faban, JMeter, and PCM.

**Fig. 21:**
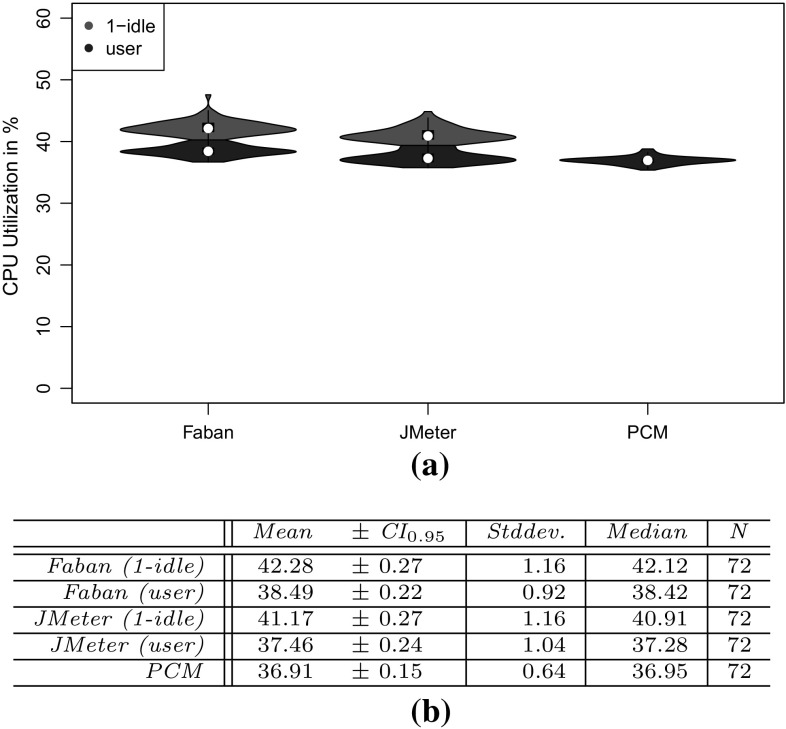
1200U-34B/33P/33M: CPU utilization statistics. **a** Violin plots. **b** Summary statistics

The CPU utilization using the extracted workload decreases to 41.17 % (1-idle) compared to the first scenario. The CPU utilization is very similar to the original workload 42.28 % (see Fig. 21). The prediction error of PCM increases to 12.7 % compared to the overall utilization and 4.1 % compared to the user CPU utilization. The response times are almost the same as in the first scenario and are therefore not shown (but are included in the supplementary material).

As a result, we can see that the workload and performance characteristics of the extracted workload and the simulated workload are comparable to the original workload when settings are changed in terms of Workload Intensity and Behavior Mix.

#### Impact of guards and actions

In the previous experiments the GaAs do not have a high impact on the workload characteristics. This results from the fact that the transactions of SPECjEnterprise2010 are designed in a way that no invalid user sequences are allowed. The only invalid user sequence can occur in the Purchase transaction. In this transaction, more items can be removed from the shopping cart than items have previously been added to the shopping cart.

Thus, we manually modify the Purchase transaction in a way that it represents a more challenging scenario. In order to achieve this, we added artificial sessions to the session log extracted with 800 users (50 % B, 25 % M, 25 % P). These sessions contain adapted sequences of user requests. During these user requests the *purchase cart* request is never called when the shoppingcart is empty. These sessions comprise three new transitions: from *view_item_quantity* to *shoppingcart*, from *shoppingcart* to *purchase cart*, and from *shoppingcart* to *defer order*. Afterward, we generated a new WESSBAS-DSL instance, again including the learning of GaAs and the calculation of the conditional probabilities (see Sect. 4.5). The resulting *Modified Purchase (MP)* transaction can be found in Fig. 12. As the GaAs do not have any impact on the Browse and Manage transactions, we set the proportion of Modified Purchase to 100 %. Afterward, a new JMeter Test Plan is generated.

To validate the impact of the automatically learned GaAs, we execute two experiments with JMeter:

1. *withGAA*: The workload is executed with the new settings using GaAs and calculated conditional probabilities (see Sect. 4.5).
2. *withoutGAA*: The guards and action are removed and the originally measured transition probabilities are included. Then, the experiment is executed again.

*Request counts* The request counts of the experiments are depicted in Table 9. The relative frequencies of the requests are almost exactly the same for the two experiments *withGAA* and *withoutGAA*. This indicates that the combination of GaAs and conditional probabilities lead to the same request count distribution.

We also included the request counts that would result when experiment *withGAA* is executed using the originally measured transition probabilities (*withGAA (OP)*) and not the conditional probabilities. In this case, the request counts would be considerably different from the originally measured request counts. Especially, the proportion of requests to *add to cart* is with 21.9 % considerably different from the originally measured proportion (16.2 %). The results of this experiment emphasize that the calculation of conditional probabilities is required.

**Table 9:** 800U-0B/100MP/0M: Request count statistics (JMeter)

	*Request*	*withGAA*		*withoutGAA*		*withGAA (OP)*	
1	Add to cart	38,596	0.162	38,720	0.162	57,762	0.219
2	Clear cart	1188	0.005	1224	0.005	946	0.004
3	Defer order	8571	0.036	8582	0.036	9682	0.037
4	Home	30,079	0.126	30,251	0.126	30,048	0.114
5	Login	30,079	0.126	30,251	0.126	30,048	0.114
6	Logout	30,079	0.126	30,251	0.126	30,048	0.114
7	Purchase cart	21,508	0.090	22,137	0.092	20,212	0.077
8	Remove	155	0.001	168	0.001	122	0.000
9	Shopping cart	21,923	0.092	21,669	0.090	17,064	0.065
10	View items quantity	56,191	0.236	56,328	0.235	68,629	0.260

**Fig. 22:**
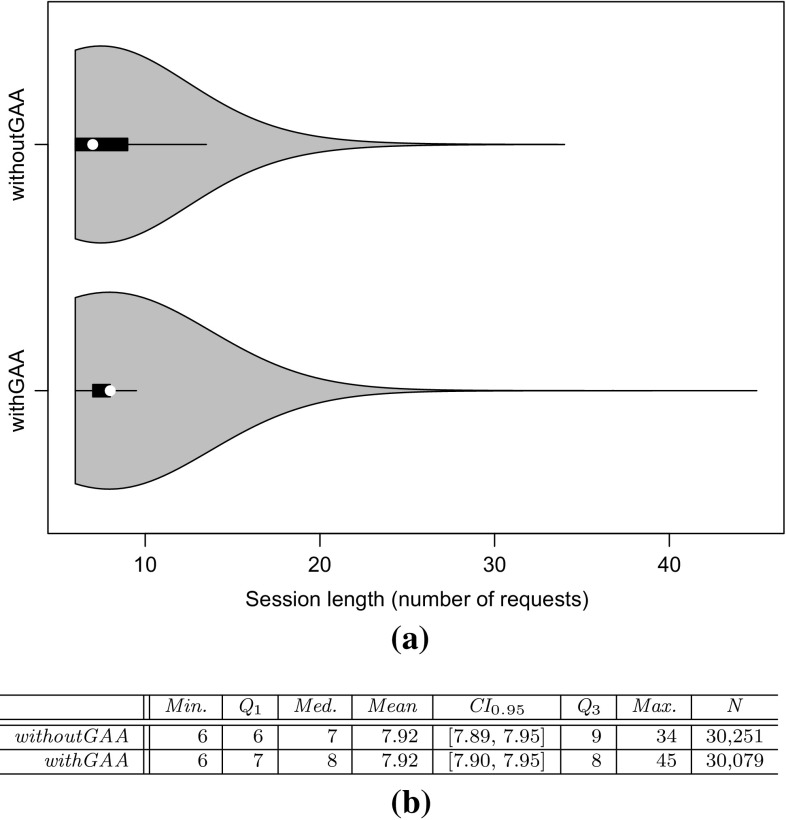
800U-0B/100MP/0M: Session length statistics for withGAA compared to withoutGAA. **a** Violin plots for session lengths. **b** Summary statistics of session lengths

*Session-based metrics* As we can see in Fig. 22, the mean session length of *withGAA* match with 7.92 exactly the mean session length of *withoutGAA*. The main difference is the number of distinct sessions. The experiment *withGAA* resulted in 182 distinct sessions and *withoutGAA* resulted in 333. This can be explained because in *withoutGAA* invalid sequences like *login*, *view items quantity*, *shopping cart*, *purchase cart* occur quite often. The GaAs prevent that a *purchase cart* request is called without having called *add to cart* before.

**Fig. 23:**
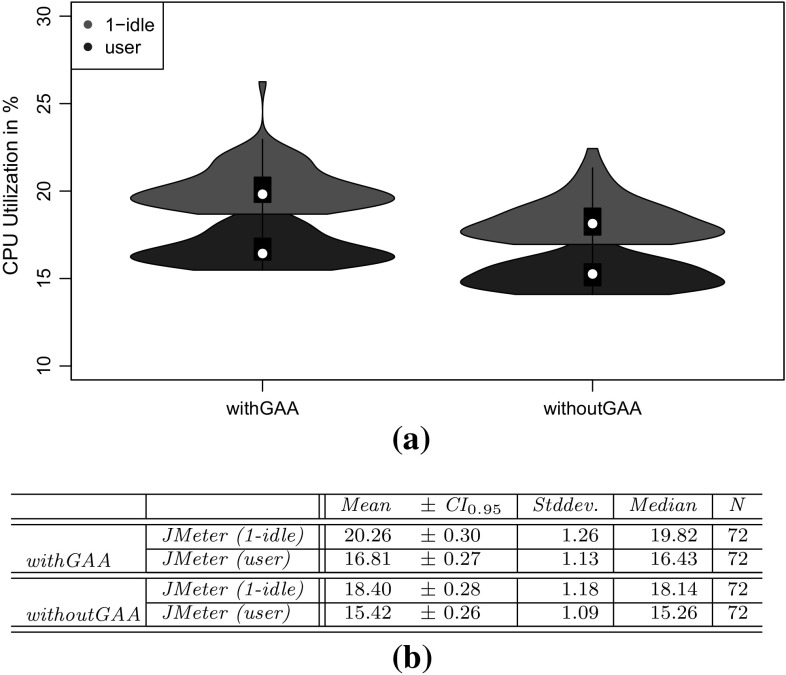
800U-0B/100MP/0M: CPU utilization statistics. **a** Violin plots. **b** Summary statistics

*CPU utilization* Figure 23 illustrates that the mean overall CPU utilization of *withoutGAA* generated by JMeter decreases by 9.18 % from 20.26 % *withGAA* to 18.40 %. This can be explained as in the experiment *withoutGAA* the action *purchase cart* is often called when no items are in the shopping cart.

**Fig. 24:**
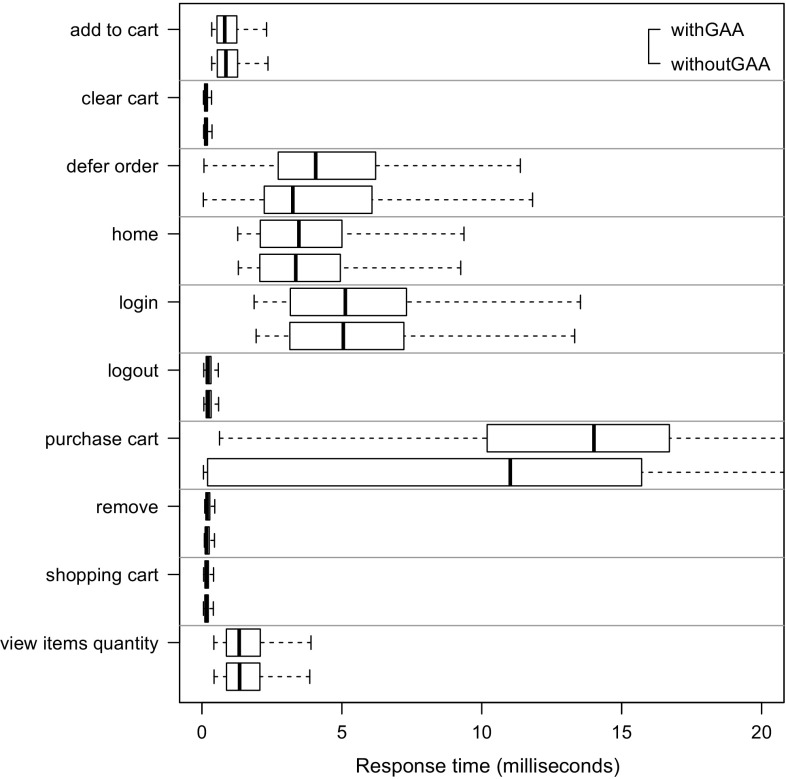
800U-0B/100MP/0M: Server-side response time statistics

*Server-side response times* The results of the server-side response times (see Fig. 24) confirm the results so far. The response times of the request type *defer order* and *purchase cart* are higher in the experiment *withGAA*. Especially, the mean response time of *purchase cart* increased significantly from 10.5 ms *withGAA* to 14.7 ms *withoutGAA*.

To summarize, the GaAs can have a high impact on performance evaluation results, depending on the control flow of the user actions. Using the conditional probabilities in combination with the GaAs the workload characteristics are similar to the originally measured workload characteristics. Only the number of distinct sessions is lower as invalid user sequences are not possible. We evaluated the impact of the GaAs only against JMeter as the performance model generator only uses average CPU values per request type and does not consider parametric dependencies like the number of items in the shopping cart. This results in the fact that the simulated CPU demands are the same regardless of whether items are in the shopping cart or not. Therefore, using a PCM model that considers this parametric dependencies would result in similar results.

### Threats to validity

A threat to external validity [57] is that we only selected one common load generator tool and one architecture-level performance evaluation tool for the evaluation. We claim that we can use the WESSBAS-DSL for other performance evaluation tools as well, which enable the specification of probabilistic workloads and GaAs. It might be that extensions of these tools are required, as described in the case of JMeter. In our future work, we will evaluate the use of other tools as well.

Another threat to validity is that we modified the dealer driver of SPECjEnterprise2010 whereby we assigned (see Sect. 7.3.2), exactly one session to a transaction. In real-world applications, users will usually not behave in this way. Instead, users will execute multiple transactions in one session, they will leave sessions without logging out of the system, or they take long breaks between user actions and reach session time outs. However, the way we split the transactions into sessions, we are able to evaluate the impact of different clustering settings on the accuracy of the results. This way we found out that X-means is easier to use than K-means and that NED is better suited to identify different transactions than ED. To overcome this threat, we also applied the clustering setting with the lowest classification error to the non-synthetic access logs of the World Cup 1998 Web site. Hence, we could show that the WESSBAS approach can also be applied to real-world application.

A threat to construct validity is that our selected workload settings do not drive the SUT in overload situations. We chose moderate CPU utilization between 30 and 50 % as in many production systems CPU utilizations are often in this moderate load situation.

To ensure conclusion validity we used multiple statistical metrics like absolute counts, relative proportions, means, medians, and standard deviations. Furthermore, we used violin and bar plots to visualize the distribution of the measurement results. As in the example in Fig. 13c, the mean values can be similar but the deviations significantly differ from each other.

As we introduced the concept of GaAs in the workload model, the memoryless property of the Markov Chains is lost. Therefore, to ensure that the average behavior extracted from the session logs is kept, we calculated and added conditional probabilities to the Behavior Models. In the evaluation of Sect. 7.4.6 we demonstrated that this behavior can be preserved for the SPECjEnterprise2010 workload. However, in future work it must be evaluated if this is generally applicable for all workload types.

### Assumptions and limitations

During our experiments a performance model generator is used [15] to create the system-specific parts of the performance model in an automated way. We were able to use this generator as it is designed for generating performance models for Java EE applications. Furthermore, the prediction accuracy of the generated model has previously been evaluated. This type of generator is not available for all session-based systems and performance models. Alternatively, the system-specific part must be modeled manually.

Within the Workload Model we assume that the loop counts follow a geometric distribution whenever a loop in a session is modeled using a memoryless loop exit transition (such as the number of $view\_items$ requests in the *Browse* transaction type (see Fig. 12)). Therefore, the distribution does not necessarily match to the distribution measured in the log files. Alternatively, for each possible loop within a Behavior Model the distribution would need to be determined and integrated into the Workload Model. This would improve the accuracy of the Workload Model but would make it more complex as there can be many different loops within a Behavior Model. It is also much more complex to consider the distribution for each loop during the transformation to performance evaluation tools. These tools must be able to handle different distributions. Furthermore, when distributions other than the geometric distribution should be used, these must be modeled explicitly. As future work, the effect of different distributions for loop counts on the workload and performance characteristics would be interesting to investigate.

One limitation of our approach is that still manual effort is needed to create the WESSBAS-DSL instances and executable load tests (see Fig. 1). This includes the identification of use cases during session log creation (Sect. 4.1), the handling of generated parameter values during the test case creation (Sect. 5), and, if required, the examination of preconditions (Sect. 7.4.4) to prevent inaccurate user behavior. However, using our approach, the effort to extract workload specifications and to generate load tests is significantly reduced. In the example of SPECjEnterprise2010 we only needed to add five regular expression extractors to the JMeter Test Plan to extract required parameter values generated during load generation. Furthermore, we had to create a mechanism to store the items added to the *shopping cart* in order to know which items can be removed in the *remove* action.

The effort for a user to adopt our approach is low when the performance evaluation tools Apache JMeter or PCM are used. The required log files can be also extracted using common monitoring tools or HTTP request logs from web servers. When other tools are used, first new transformations of the WESSBAS-DSL to these tools must be implemented.

Another limitation of our approach is that the order of events and the minimum and maximum number of executions is not controlled using probabilistic workloads. As we can see in Sect. 7.4.4, the number of items in the shopping cart has a high impact on the response times of the purchase cart action.

## Conclusion and future work

The specification and generation of representative workloads is a core task for many performance evaluation activities. However, obtaining representative workload specifications is still a big challenge. In response to this challenge, we present our WESSBAS approach for the systematic extraction and specification of probabilistic workloads for session-based systems. We also include transformations to the load testing tool Apache JMeter and to the performance model PCM. To address the challenge of specifying workloads for different performance evaluation tools, we first introduced a domain-specific language that describes the structure of a workload in a generalized way. We demonstrated how groups of customers with similar behavioral patterns can be identified using clustering algorithms. Furthermore, inter-request dependencies are learned in an automatic way and conditional probabilities are calculated. This is the first approach to present a holistic process from runtime data to the executable load tests and performance predictions.

The evaluation with the industry-standard benchmark SPECjEnterprise2010 and the World Cup 1998 access logs demonstrated the practicality and high accuracy of the proposed approach. The session-based characteristics, like session length and the number of distinct sessions, deviate from the measured logs in case of SPECjEnterprise2010. However, using the non-synthetic World Cup logs, the session-based characteristics are similar as well. The invocation frequencies for requests match with almost 100 %. Furthermore, performance characteristics in terms of CPU utilization, response times and heap usage are, with a few minor exceptions, similar to the original executed workload. The approach is applicable for all session-based systems and requires no detailed knowledge about workload extraction.

In our future work, we will investigate the prioritization and selection of load test cases using the generated performance models [53]. Moreover, we plan to implement the transformation between the WESSBAS-DSL instances and PCM in a bidirectional way. The advantage of testing WESSBAS-DSL instances and PCM in a bidirectional way is that the test cases are analyzed and selected within PCM and corresponding load test scripts can be generated using the WESSBAS-DSL. Furthermore, we plan to implement the transformation from WESSBAS to PCM in a transformation language such as Henshin,^8^ as it additionally provides tools to verify the transformation correctness. Moreover, we plan to integrate approaches for the generation of varying workload intensities [49].

## Appendix

See Table 10.

**Table 10:** Resulting GaAs

From state	To state	Guards	Actions
Login	View items	Login	N/A
Login	Inventory	Login	Inventory $=$ true
Login	View items quantity	Login	View_Items_quantitydeferorder $+$1; View_Items_quantitypurchasecart $+$1; View_Items_quantityshoppingcart $+$1; View_Items_quantityclearcart $+$1; View_Items_quantityremove $+$1
View items quantity	Add_to_Cart	N/A	Add_to_Cartdeferorder $+$1; Add_to_Cartpurchasecart $+$1; Add_to_Cartshoppingcart $+$1; Add_to_Cartclearcart $+$1; Add_to_Cartremove $+$1
Add_to_Cart	Defer order	Login&& View_Items_quantitydeferorder >0&& Add_to_Cartdeferorder >0	View_Items_quantitydeferorder $-1$; Add_to_Cartdeferorder $-1$
Add_to_Cart	Purchasecart	Login&& View_Items_quantitypurchasecart >0&& Add_to_Cartpurchasecart >0	View_Items_quantitypurchasecart $-1$; Add_to_Cartpurchasecart $-1$
Add_to_Cart	Shoppingcart	Login&& View_Items_quantityshoppingcart >0&& Add_to_Cartshoppingcart >0	View_Items_quantityshoppingcart $-1$; Add_to_Cartshoppingcart $-1$;
Add_to_Cart	View items quantity	Login	View_Items_quantitydeferorder $+$1; View_Items_quantitypurchasecart $+$1; View_Items_quantityshoppingcart $+$1; View_Items_quantityclearcart $+$1; View_Items_quantityremove $+$1
Shoppingcart	Clearcart	Login&& View_Items_quantityclearcart >0&& Add_to_Cartclearcart >0	View_Items_quantityclearcart $-1$; Add_to_Cartclearcart $-1$
Shoppingcart	Remove	Login&& View_Items_quantityremove >1&& Add_to_Cartremove >1	View_Items_quantityremove $-1$; Add_to_Cartremove $-1$
Remove	Defer order	Login&& View_Items_quantitydeferorder >0&& Add_to_Cartdeferorder >0	View_Items_quantitydeferorder $-1$; Add_to_Cartdeferorder $-1$
Remove	Purchasecart	Login&& View_Items_quantitypurchasecart >0&& Add_to_Cartpurchasecart >0	View_Items_quantitypurchasecart -1; Add_to_Cartpurchasecart $-1$
Remove	Shoppingcart	Login&& View_Items_quantityshoppingcart >0&& Add_to_Cartshoppingcart >0	View_Items_quantityshoppingcart $-1$; Add_to_Cartshoppingcart $-1$
Clearcart	View items quantity	N/A	View_Items_quantitydeferorder $+$1; View_Items_quantitypurchasecart $+$1; View_Items_quantityshoppingcart $+$1; View_Items_quantityclearcart $+$1; View_Items_quantityremove $+$1
Inventory	Sellinventory	Login&& Inventory	N/A
Inventory	Cancelorder	Login	N/A
Inventory	Inventory	Login	Inventory $=$ true
Cancelorder	Inventory	N/A	Inventory $=$ true
Sellinventory	Home	Login	N/A
Sellinventory	Sellinventory	Login&& inventory	N/A
View items	Home	Login	N/A
View items	View items	Login	N/A
**Additionally for RQ5**			
View items quantity	Shoppingcart	Login&& View_Items_quantityshoppingcart >0&& Add_to_Cartshoppingcart >0	View_Items_quantityshoppingcart $-1$;Add_to_Cartshoppingcart $-1$
Shoppingcart	Purchasecart	Login&& View_Items_quantitypurchasecart >0&& Add_to_Cartpurchasecart >0	View_Items_quantitypurchasecart $-1$;Add_to_Cartpurchasecart $-1$
Shoppingcart	Defer order	Login&& View_Items_quantitydeferorder >0&& Add_to_Cartdeferorder >0	View_Items_quantitydeferorder-1;Add_to_Cartdeferorder $-1$

## Footnotes

1
Wessbas is an acronym for *Workload Extraction and Specification for Session-Based Application Systems*.

2 WESSBAS tool support: https://github.com/Wessbas.

3
https://commons.apache.org/proper/commons-jexl/.

4
http://se.informatik.uni-wuerzburg.de/tools/limbo/.

5 SPECjEnterprise is a trademark of the Standard Performance Evaluation Corp. (SPEC). The SPECjEnterprise2010 results or findings in this publication have not been reviewed or accepted by SPEC, therefore no comparison nor performance inference can be made against any published SPEC result. The official Web site for SPECjEnterprise2010 is located at http://www.spec.org/jEnterprise2010.

6
http://java.net/projects/faban/.

7
http://linux.die.net/man/1/sar.

8
http://www.eclipse.org/henshin.
